# Effectiveness of hormone add-on strategies in ovarian stimulation for women with poor ovarian response: a systematic review and network meta-analysis of randomized controlled trials

**DOI:** 10.1007/s10815-025-03633-z

**Published:** 2025-10-25

**Authors:** Andrea Etrusco, Vittorio Agrifoglio, Juan Carlos Castillo Farfán, Andrea Bernabeu Garcia, Antonio Simone Laganà, Belén Moliner Renau, Antonio D’Amato, Pasquale De Franciscis, Ana Fuentes Rozalén, Gaetano Riemma

**Affiliations:** 1 Department of Reproductive Medicine. Instituto Bernabeu, Alicante, 03016 Spain; 2 Department of Health Promotion, Mother and Child Care, Internal Medicine and Medical Specialties (PROMISE), University of Palermo, 90133 Palermo, Italy; 3 Catedra de Medicina Comunitaria y Salud Reproductiva, Universidad Miguel Hernandez, Elche, 03202 Spain; 4 Unit of Obstetrics and Gynecology, “Paolo Giaccone” Hospital, Palermo, 90127 Italy; 5 Unit of Obstetrics and Gynecology, Department of Interdisciplinary Medicine (DIM), University of Bari “Aldo Moro”, Policlinico of Bari, Piazza Giulio Cesare 11, Bari, 70124 Italy; 6 Department of Woman, Child and General and Specialized Surgery, University of Campania “Luigi Vanvitelli”, Largo Madonna delle Grazie 1, Naples, 80138 Italy

**Keywords:** Diminished ovarian reserve, Poor responders, Assisted reproductive technologies, IVF, Infertility

## Abstract

**Purpose:**

Despite various stimulation protocols and adjuvant treatments, optimizing the reproductive success of women with poor ovarian response (POR) undergoing assisted reproduction techniques (ART) remains a challenge. The aim was to evaluate and compare the ovarian stimulation and reproductive outcomes of hormonal add-ons in women with POR undergoing ART.

**Methods:**

MEDLINE, LILACS, SCOPUS, EMBASE, Scielo.br, PROSPERO, CINAHL, PsycINFO, AMED, Clinicaltrials.gov, ICTRP, Cochrane Library, and conference proceedings were searched for randomized controlled trials without temporal, geographic, and language restrictions. We included only RCTs that allocated women with POR, according to Bologna criteria, undergoing ART to at least two different ovarian stimulation protocols, with the treatment arm including at least one hormonal add-on. A random-effect network meta-analysis was performed for mixed multiple treatment comparisons to rank available add-ons by the surface under the cumulative ranking curve area (SUCRA). Primary outcome was the live birth rate.

**Results:**

Twenty-two studies (4131 women) were directly and indirectly compared. Concerning the live birth rate, no significant differences among add-ons were noted (very low evidence), with testosterone (SUCRA = 34.0%) showing the highest probability of the best treatment. For the clinical pregnancy rate, according to SUCRA ranking, human growth hormone (SUCRA = 46.3%) and testosterone (SUCRA = 44.6%) had increased chances of being ranked first, with growth hormone being significantly more efficacious than estrogens (OR 3.46 [95% CI 1.59 to 7.53]; low evidence) while recombinant luteinizing hormone was significantly less efficacious (OR 0.50 [95% CI 0.26 to 0.96]; very low evidence). Regarding the overall number of retrieved oocytes and the mean number of metaphase II oocytes, human growth hormone was confirmed best ranked (SUCRA = 53.2% and SUCRA = 67.9%). Letrozole had significantly less gonadotropins used than controls (SMD – 7.02 [95% CI − 12.82 to − 1.22]; low evidence) (SUCRA = 67.0%) and the smallest stimulation duration (SUCRA = 52.0%).

**Conclusion:**

Low to very-low evidence shows that women with POR undergoing controlled ovarian stimulation may benefit from adding human growth hormone or testosterone for improved reproductive outcomes. However, additional high-quality randomized controlled trials are needed to overcome the limitations of the current literature.

Trial registration: PROSPERO CRD42024618797, date: 25 November 2024.

**Supplementary Information:**

The online version contains supplementary material available at 10.1007/s10815-025-03633-z.

## Introduction

Ovarian reserve refers to the number of oocytes a woman has at a given stage of her life. Poor ovarian response (POR) is due to a decline in the number of oocytes resulting in inadequate ovarian function and reduced fertility [1]. This condition is typically characterized by decreased levels of anti-Müllerian hormone (AMH), decreased antral follicle number (AFC), and sometimes also elevated follicle-stimulating hormone (FSH) at baseline [2]. Some pieces of evidence report that the decline in number is also often accompanied by a concomitant decline in oocyte quality [3]. In fact, it is debated whether the natural decline in oocyte quantity is related to a decrease in oocyte quality and an increased risk of aneuploidies [4]. In support of this hypothesis, data suggest that women with POR are at higher risk of aneuploid pregnancies and miscarriages [5, 6].Therefore, compromised reproductive potential characterizes women with POR undergoing ovarian stimulation (OS) and assisted reproductive technologies (ART) compared with comparably aged counterparts with normal ovarian reserve and OS responsiveness [7]. However, the ideal stimulation regimen for patients with POR is currently unknown. In fact, several therapeutic strategies have been proposed to manage women with POR, seeking to increase the number of good-quality oocytes collected following OS and to optimize ART outcomes [8–10]. Among these, several stimulation protocols, with or without pre-treatment, and involving the use of various hormonal [11–15], pharmacological [16, 17] and non-pharmacological [18–20] add-ons to gonadotropins have been described in numerous randomized controlled trials (RCTs). In fact, a recent meta-analysis of RCTs suggested that pretreatment with testosterone could improve the live birth rate (LBR) in women with (POR) [21]. However, this conclusion is derived from heterogeneous comparisons that include different treatment protocols, making the interpretation of the results difficult. To date, there is only one analysis that attempts such a multiple comparison to evaluate adjuvant treatments in OS for poor responder women in IVF cycles, but it has important methodological limitations and doubts about the quality and appropriateness of the included studies [22].Therefore, given these assumptions, to date the superiority of one protocol with one add-on over another has not yet been demonstrated.This systematic review and network meta-analysis aims to explore the superiority of an OS protocol with hormonal add-ons over the same protocol without additions in terms of reproductive and ART outcomes in women with POR according to the Bologna criteria [23], analyzing currently available randomized controlled trials conducted for this purpose.

## Methods

This network meta-analysis was carried out using the procedures described in the Cochrane Handbook for Systematic Reviews of Interventions [24] and the Mbuagbaw et al. methodological specifications [25]. The Preferred Reporting Items for Systematic Reviews and Meta-Analyses (PRISMA) extension statement for network meta-analyses (PRISMA-NMA) was adhered to.The study protocol (CRD42024618797) was submitted to the PROSPERO (International Prospective Register of Systematic Reviews) database on 25 November 2024.

### Data sources and search strategy

The following Medical Subject Heading (MeSH) terms and keywords were used to search the electronic databases MEDLINE (available through PubMed), SCOPUS, LILACS, EMBASE, Scielo.br, and PROSPERO: “ovarian stimulation”/“ovulation induction” (MeSH Unique ID: D010062), “assisted reproductive technologies” (MeSH Unique ID: D027724), “in vitro fertilization” (MeSH Unique ID: D005307), “reproductive techniques”, “POSEIDON criteria”, “Bologna criteria”, “low responders”, “poor responders”, “poor ovarian response”, “poor response”, “poor ovarian responsiveness”, “ovarian reserve” (MeSH Unique ID: D065851), “diminished ovarian reserve”, “poor ovarian response”and “poor ovarian reserve”. without any date restrictions. The search query was changed to fit the format of each database (Appendix 1). A filter for randomized controlled trials (RCTs) was used in each database.Additional searches were conducted to find more relevant papers on CINAHL, PsycINFO, and AMED in order to reduce publication bias. The Cochrane Central Register of Controlled Studies, Clinicaltrials.gov, and the World Health Organization’s International Clinical Trials Registry Platform (ICTRP) were also searched to locate additional randomized controlled trials. Additionally, the gray literature (NTIS, PsycEXTRA) was examined to find national and international conference abstracts. The included study’s references and pertinent reviews were also reviewed to locate more articles that eluded our first search.The analysis did not include any editorials, letters to the editor, comments, or second thoughts publications. A.E. and V.A. carried out the approach for the literature search.

### Data collection and study selection

The titles and abstracts of all the papers were separately screened by two reviewers (G.R. and J.C.C.F.) to identify which research should be evaluated further and to omit citations that were judged unnecessary by both observers.The authors, institutions, publication titles, and study findings were all concealed during this initial screening. Any ambiguity or disagreement was cleared up by talking with a third reviewer (A.F.R). Two reviewers (A.B.G. and B.M.R.) retrieved and evaluated full texts of possibly relevant articles based on the pre-established inclusion criteria. In addition, methodological validity was evaluated before being included in the review. Every doubt or disagreement was cleared up by discussion among reviewers in order to reach an agreement.Add-ons have been defined as any additional treatment used, in addition to gonadotropin releasing hormone (GnRH) analogs and gonadotropins, during the vitro fertilization (IVF)/intracytoplasmic sperm injection (ICSI) cycle, with the goal of increasing pregnancy success in women with POR. As such, hormonal add-ons directly affect the endocrine system and influence ovarian response, oocyte quality, or endometrial receptivity. In this review, we include letrozole (LTZ) and clomiphene citrate (CC) as hormonal add-ons, although they are more commonly defined as ovulation-inducing agents. We included only RCTs that allocated women with POR, according to Bologna criteria [23], undergoing ART to at least two different OS protocols with the treatment arm including at least one hormonal add-on. We chose to use the Bologna criteria to strictly limit the inclusion criteria of patients with POR in the network meta-analysis. Considering the recent introduction but the paucity of studies adopting exclusively or predominantly the POISEDON criteria, we opted to exclusively use the Bologna criteria in the present analysis to ensure greater homogeneity of the population, significantly reducing clinical and methodological variability and thus increasing the reliability and generalizability of the results thanks to their validation and consolidation in a wider amount of RCTs.

Control arms consisted of women undergoing the same OS protocol without the hormonal add-ons.

The exclusion criteria were the following: studies including different OS protocols between treatment and control groups, studies with women not matching the Bologna criteria [23] and studies evaluating non-hormonal add-ons defined as additional treatments that improve metabolic, immunologic, and supportive factors without directly modifying hormonal balance.

### Data extraction

The extraction forms were made especially for this network meta-analysis.

The nation, study design, study type, inclusion and exclusion criteria, study period, location, were extracted from each study. For each group (OS + add-ons and OS without add-ons) the following study characteristics were gathered in order to characterize the included studies: factors such as the number of women, the women’s age, body mass index (BMI), the type and duration of her infertility, the AFC, the AMH, day-3 FSH, the number of previous failed fertility treatments, the cause of infertility besides poor ovarian response, the method of diagnosis, the characteristics of IVF/ICSI, the protocol for OS, the duration of the stimulation, the total dosage of gonadotropins used during the OS, the cancelation rate, the number of retrieved and metaphase II (MII) oocytes, the rates of fertilization, the clinical pregnancy rate (CPR) and the LBR.

Each abstract was examined and categorized independently by two authors (A.E. and V.A.). In order to agree on possible application, the same two writers carefully read the texts of the selected studies and independently gathered pertinent information about the research characteristics and the noteworthy findings.

After discussing each contradiction with the other three authors (J.C.C.F., A.F.R. and B.M.R.), the reviewers came to a consensus. Unpublished data were acquired by contacting the original study authors directly when the study methods specified that additional outcome data were collected.

### Assessment of risk of bias

The risk of bias for the included RCTs was assessed using the Cochrane Risk of Bias Tool version 2 (RoB 2.0) [26]. This tool categorizes the risk of bias in research into five areas: the randomization process, deviations from intended interventions, missing outcome data, outcome evaluation, and choice of reported result. Three risk levels are evaluated for every domain: “low risk”, “some concerns” or “high risk”.

Three authors (A.E., V.A. and G.R.) independently evaluated the risk of bias assessment. The dispute was settled after concealment with J.C.C.F., A.F.R. and A.B.G.

We examined the feasibility and comparability of baseline patient characteristics, the reported results, and the registration of trials before the recruitment of the first patient (for RCTs that began less than 15 years ago) to further assess the reliability of the studies. Additionally, we examined each study’s compliance with the CONSORT declaration and any believable retractions or expressions of concern using the PubMed and RetractionWatch.org databases. Furthermore, the Confidence in Network Meta-Analysis framework (CINeMA) criteria were used to evaluate the certainty of the evidence [27]. Publication bias was assessed using funnel plots and Egger’s test for each outcome reported in at least 10 trials.

### Outcomes

The primary outcome of the present network meta-analysis was the LBR, where a live birth was defined according to the International Glossary on Infertility and Fertility Care [28] as the complete expulsion or removal of a fetus from a woman after at least 22 weeks’ gestation, which, when separated, manifests vital signs.

Secondary outcomes were the CPR, defined as pregnancy diagnosed by ultrasonographic visualization of one or more gestational sacs or definitive clinical signs of pregnancy [28]; total dosage of gonadotropins used during the OS; duration in days of the OS; the serum estrogen levels at the day of trigger with the human chorionic gonadotropin (hCG) or the agonist of the GnRH; the number of oocytes retrieved; the number of MII oocytes retrieved; the fertilization rate, defined as the number of fertilized oocytes on the total number of oocytes; the cancellation rate defined as the number of cycles cancelled due to ultrasonographic non-response to gonadotropin administration or absence of oocytes obtained out of the number of oocyte retrievals.

### Data synthesis

All data analysis and graphical representations were done using STATA version 14.1 (StataCorp, College Station, TX).

The network assumption of overall consistency was statistically confirmed for every meaningful result using the < network meta consistency > command. The local test on loop inconsistencies was then carried out using the Separating Indirect from Direct Evidence (SIDE) procedure with the command < network sidesplit all >. When no discrepancy between the global and local testing findings was found, the consistency assumption was accepted. The study’s direct and indirect comparisons would undoubtedly yield pertinent results in this case, as the consistency model demonstrated that any differences in the results were only attributable to chance mistakes and the effect of the intervention. The summary measurements were presented as odds ratios (OR) for categorical variables and standardized mean differences (SMD) for continuous variables with 95% confidence intervals (CI) using the Der Simonian and Laird random effects model. A Higgins I^2^ score higher than 25% suggested the possibility of heterogeneity. To evaluate the efficacy of the different hormonal add-ons and rank the therapies to determine which was statistically better, a forest plot and an interval plot together with a Surface Under the Cumulative Ranking curve Area [SUCRA] ranking plot were made for each outcome under study.

## Results

**Fig. 1:**
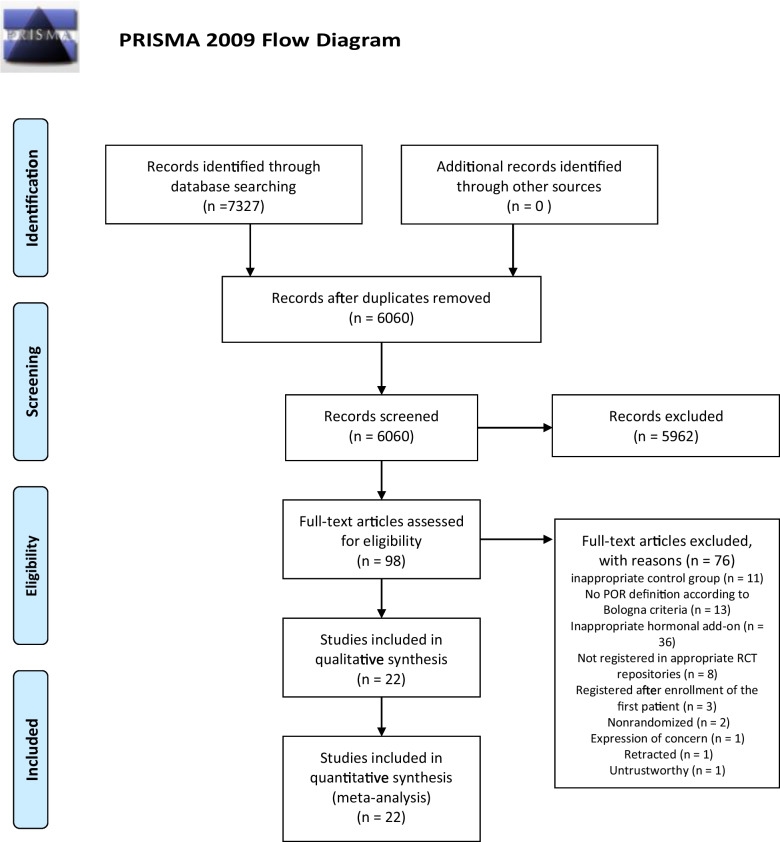
PRISMA flowchart of included studies in systematic review and network meta-analysis

A total of 7327 studies were initially identified using database searches. Of those, 1267 were removed as duplicates. After title and abstract screening, 5962 papers were subsequently removed (Fig. 1). Ninety-eight papers underwent Full-text assessment, of which 36 studies [10, 18–20, 29–59] were excluded for not having an appropriate hormonal add-on, 13 studies [14, 15, 60–70] for not respecting the POR definition according to Bologna criteria [23], 11 studies [9, 71–80] were excluded for inappropriate control group, 8 studies for not being registered in appropriate RCT repositories, 3 studies [81–83] for being registered after enrollment of the first patient, 2 studies [84, 85] for being non-randomized, 1 study [86] for having an expression of concern, 1 study [87] for being retracted, and 1 study [88] for untrustworthiness for the criteria mentioned above (Supplementary Table 1) (Fig. 1).

Therefore, 22 studies [12, 13, 16, 17, 89–106] were included in the systematic review and network meta-analysis (Fig. 1).

The potential benefits of the following protocols with hormonal add-ons were analyzed: addition of LTZ [16, 95, 97], addition of CC [17, 98, 100], addition of recombinant luteinizing hormone (rLH) [94, 102, 104], pretreatment with estrogen [13], pretreatment with dehydroepiandrosterone (DHEA) [92, 103, 105, 106], pretreatment with testosterone [89, 91, 93, 99, 101], and addition of human growth hormone (GH) [12, 90, 96].

### Study characteristics

The main characteristics of the included studies are summarized in Table 1, and the baseline patients’ characteristics are presented in Table 2.

**Table 1:** Main characteristics of studies included in network meta-analysis

Study	Primary outcome(s)	Add-ons	Inclusion criteria	Exclusion criteria	Nationality	Trial ID
Aflatoonain, 2022	Clinical and biochemical pregnancy rates	Addition of testosterone	Poor ovarian response based on Bologna criteria	• Endocrine disorders • Intrauterine disorders • Azoospermia • Severe endometriosis	Iran	IRCT20180818040828N1
Bastu, 2016	Number of oocytes retrieved	Addition of letrozole	Poor ovarian response based on Bologna criteria	• History of chemotherapy/radiotherapy • ovarian surgery • DHEA/testosterone use	Turkey	NCT02293668
Bayoumi, 2016	Mean numbers of mature oocytes retrieved and fertilized	Addition of Growth Hormone	Poor ovarian response based on Bologna criteria	• FSH > 20 IU/L, previous ovarian surgery • Non-POR infertility causes • PCOS • Endocrine disorders • Male factor infertility	Egypt	NCT02185326
Bosdou, 2016	Number of COCs retrieved	Addition of testosterone	Poor ovarian response based on Bologna criteria	• BMI ≥ 32 kg/m2 • Endometriosis stage III-IV • Ovarian surgery • Endocrine/metabolic disorders • Use of testicular sperm	Greece	NCT01961336
Fu, 2017	High-quality embryo yield	Addition of DHEA	Poor ovarian response based on Bologna criteria	• Endometriosis • History of chemotherapy • Ovarian surgery • Prior DHEA use	China	NCT02866253
Hoang, 2021	Number of mature oocytes retrieved	Addition of testosterone	Poor ovarian response based on Bologna criteria	• POI • Male factor infertility • Severe endometriosis • Thyroid/liver/kidney disease • Abnormal genitalia	Vietnamese	NCT04602143
Humaidan, 2017	Number of oocytes retrieved	Addition of rLH	Poor ovarian response based on Bologna criteria	• Women aged ≥ 41 years • POI • Preimplantation genetic screening	Denmark	NCT02047227
Liu, 2020	Cumulative Live Birth Rate	Addition of Letrozole	Poor ovarian response based on Bologna criteria	• Repeated IVF failure • Severe endometriosis • PCOS	China	ChiCTR-TRC-13003454
Moffat, 2021	Number of oocytes retrieved	Addition of Clomiphene Citrate	Poor ovarian response based on Bologna criteria	N/D	Switzerland	NCT01577472
Mohammad, 2020	Clinical pregnancy rate	Addition of Growth Hormone	Poor ovarian response based on Bologna criteria	• Known medical disease (e.g. severe hypertension or hepatic disease) • History of altered karyotype in one or both partners • History of chronic, autoimmune or metabolic diseases • Presence of endocrinopathies • Male factor infertility	Egypt	NCT03759301
Moini, 2019	number of oocytes retrieved and the number of oocytes MII	Addition of Letrozole	Poor ovarian response based on Bologna criteria	• POI • Donor/recipient treatments • Metabolic or endocrine disorders including hyperprolactinemia and hypo/hyperthyroidism • Endometriosis • Body mass index (BMI) > 30 kg/m2 • Azoospermic male partner	Iran	IRCT201701291952N8
Norman, 2019	Live birth rate	Addition of Growth Hormone	Poor ovarian response based on Bologna criteria	• Any clinically significant systemic disease, • History of radiotherapy or chemotherapy • Any current history of malignant disease • Pituitary or hypothalamic disease • Current ovarian cyst > 3 cm • Any chronic infectious diseases • PCOS • Unexplained menstrual bleeding • Preimplantation genetic testing • Smokers • Using steroids, DHEA or prednisolone in the last 3 months	Australia	ACTRN12609001060235
Ragni, 2012	delivery rate per started cycle	Addition of Clomiphene Citrate	Poor ovarian response based on Bologna criteria	• Number of previous IVF cycles ≥ 3 • Cycles requiring the use of spermatozoa from MESA-TESE procedures	Italy	NCT01389713
Saharkhiz, 2018	mean number of oocytes and embryo	Addition of Testosterone	Poor ovarian response based on Bologna criteria	• Presence of endocrine disorders (thyroid, prolactin, etc.) • Presence of endometrioma and any history of surgery on the ovaries • Reluctance to participate in the project • New clinical conditions or a change in a treatment procedure • Sensitivity to testosterone gel	Iran	IR.SBMU.RETECH.REC.1395.1007
Siristatidis, 2017	Number of Cumulus- oocyte complexes retrieved	Addition of Clomiphene Citrate	Poor ovarian response based on Bologna criteria	• Basal level of FSH at day 3 of menstrual cycle > 20 IU/l) • Increased BMI > 35 kg/m2 • History of endocrine or metabolic disorders • History of ovarian cystectomy or oophorectomy • Severe endometriosis • Severe azoospermia	Greece	NCT01319708
Subirà, 2021	Number of MII oocytes	Addition of Testosterone	Poor ovarian response based on Bologna criteria	• Non-corrected uterine malformations, endometrial pathology • Severe male factor (motile sperm count < 1) • Hydrosalpinx • Premature ovarian insufficiency, body mass index (BMI) > 35 kg/m2 • Androgen treatment within the last 3 months • Known allergy to the experimental drug	Spain	NCT03378713
Tosun, 2021	GCs apoptosis rate in terms of viability, early apoptosis, late apoptosis and necrosis	Addition of rLH	Poor ovarian response based on Bologna criteria	• Male factor • POI • Need of preimplantation genetic testing	Turkey	NCT03527823
Wang, 2022	Live birth rate	Addition of DHEA	Poor ovarian response based on Bologna criteria	• Women that failed to obtain clinical pregnancy after three or more IVF/ICSI cycles • History of two or more recurrent pregnancy losses • Diagnosis of uterine abnormalities using hysteroscopy • Diagnosed with hydrosalpinx using hystero salpingography • History of chemotherapy with cytotoxic agents • History of pelvic radiotherapy • Epilepsy; • Treatment with DHEA before study enrolment • Allergy to DHEA	China	ChiCTR-IPR-15006909
Yeung, 2014	AFC after 12 weeks of treatment	Addition of DHEA	Poor ovarian response based on Bologna criteria	• History of ovarian cystectomy or oophorectomy • History of cytotoxic chemotherapy • History of pelvic irradiation • History of taking testosterone or DHEA supplementation	Hong Kong	NCT01915186
Younis, 2016	maximal serum E2 level on the day of hCG administration	Addition of rLH	Poor ovarian response based on Bologna criteria	• Severe endometriosis • Uncontrolled thyroid disease • Diabetes mellitus • Significant hyperprolactinaemia • Hypogonadotrophic hypogonadism • More than four previous unsuccessful ART attempts	Israel	NCT01016210
Zhang, 2014	follicular fluid BMP- 15, GDF-9 and serum AMH, FSH, E_2_	Addition of DHEA	Poor ovarian response based on Bologna criteria	• History of ovarian cystectomy or oophorectomy • Diagnosis of endometriosis • History of DHEA supplementation or hormonal replacement therapy	China	ChiCTR-TRC-14005002
Zhang, 2022	total number of retrieved oocytes	Addition of Estrogen	Poor ovarian response based on Bologna criteria	• Preimplantation genetic testing cycle • Donor cycle • Family history of thrombosis or a high risk of thrombosis • Previous history of hypertension or hypertension • Hyperlipidemia • Estrogen-dependent breast disease • Cervical biopsy showing cervical intraepithelial neoplasia grade III or above	China	NCT03300518

*PCOS* polycistic ovary syndrome, *POI* premature ovarian insufficiency, *BMI* body mass index, *FSH* follicle-stimulating hormone, *rLH* recombinant lutenizing hormone, *DHEA* Dehydroepiandrosterone, *MII* metaphase II, *MESA* microsurgical epididymal sperm aspiration, *TESE* testicular epididymal sperm extraction, *AMH* anti-Mullerian hormone, *BMP*−15 bone morphogenetic protein-15, *GDF-9* growth differentiation factor-9, *E2* estradiol

**Table 2:** Baseline patient characteristics

Study	Group	Age (mean ± SD), years	AMH (mean ± SD), ng/ml	AFC (mean ± SD), *n*	Previously cycles with POR
Aflatoonain, 2022	Control	36.21 ± 3.01	0.82 ± 0.08	5.54 ± 2.13	N/D
	Addition of Testosterone	35.38 ± 5.23	0.88 ± 0.08	5.34 ± 1.69	N/D
Bastu, 2016	Control (450 IU)	36.94 ± 3.33	0.55 ± 0.35	4	N/D
	Control (300 IU)	35.00 ± 3.10	0.71 ± 0.28	3	N/D
	Addition of Letrozole	37.52 ± 4.06	0.64 ± 0.41	4	N/D
Bayoumi, 2016	Control	34.8 ± 5.6	0.5 ± 0.2	5.9 ± 1.7	2.7 ± 1.5
	Addition of Growth Hormone	34.9 ± 4.8	0.4 ± 0.2	5.9 ± 1.6	2.4 ± 1.5
Bosdou, 2016	Control	42.5 ± 4.0	0.65 ± 0.78	5.0 ± 3.0	N/D
	Addition of Growth Hormone	41.5 ± 3.0	0.97 ± 0.65	6.0 ± 4.0	N/D
Fu, 2017	Control	36.8 ± 4.3	1.00 ± 0.77	3.57 ± 1.16	N/D
	Addition of DHEA	37.4 ± 3.6	1.01 ± 0.61	3.74 ± 1.43	N/D
Hoang, 2021	Control	36.4 ± 5.2	1.0 ± 0.3	5.9 ± 3.6	N/D
	Addition of Testosterone (4 weeks)	35.9 ± 5.4	1.0 ± 0.4	5.4 ± 2.6	N/D
	Addition of Testosterone (6 weeks)	36.2 ± 4.7	0.9 ± 0.4	4.9 ± 1.9	N/D
Humaidan, 2017	Control	38.3 ± 3.0	0.60 ± 0.48	4.8 ± 2.2	84.3%
	Addition of rLH	38.3 ± 2.9	0.58 ± 0.50	4.9 ± 2.3	82.0%
Liu, 2020	Control	39 ± 1.5	0.8 ± 0.1	5 ± 0.33	N/D
	Addition of Letrozole	37 ± 1.17	0.6 ± 0.1	4 ± 0.33	N/D
Moffat, 2021	Control (450 IU)	38.8 ± 1.3	0.78 ± 0.15	5.5 ± 1.2	N/D
	Control (150 IU)	39.9 ± 0.6	0.66 ± 0.09	6.0 ± 1.0	N/D
	Addition of Clomiphene Citrate (450 IU)	38.4 ± 1.0	0.64 ± 0.12	6.0 ± 0.5	N/D
	Addition of Clomiphene Citrate (150 IU)	39.9 ± 1.2	0.84 ± 0.14	6.5 ± 0.8	N/D
Mohammad, 2020	Control	34.7 ± 2.0	0.69 ± 0.16	5.8 ± 1.8	2.6 ± 0.3
	Addition of Growth Hormone	34.3 ± 2.4	0.72 ± 0.09	5.7 ± 1.8	2.5 ± 0.2
Moini, 2019	Control	36.5 ± 3.7	0.73 ± 0.31	4.8 ± 1.5	1.1 ± 0.6
	Addition of Letrozole	37.2 ± 3.3	0.75 ± 0.35	5.1 ± 2.0	1.0 ± 0.9
Norman, 2019	Control	N/D	N/D	N/D	N/D
	Addition of Growth Hormone	N/D	N/D	N/D	N/D
Ragni, 2012	Control	38.5 ± 3.1	0.76 ± 1.0	3.9 ± 2.6	N/D
	Addition of Clomiphene Citrate	38.6 ± 2.9	0.71 ± 1.0	3.4 ± 2.5	N/D
Saharkhiz, 2018	Control	39.7 ± 3.3	0.6 ± 0.5	3.1 ± 2.6	N/D
	Addition of Testosterone	41.0 ± 3.8	0.5 ± 0.6	2.2 ± 0.6	N/D
Siristatidis, 2017	Control	40.0 ± 3.0	N/D	4.0 ± 2.2	N/D
	Addition of Clomiphene Citrate	39.0 ± 2.7	N/D	3.0 ± 1.3	N/D
Subirà, 2021	Control	35.2 ± 3.0	0.5 ± 0.3	N/D	0.8 ± 0.4
	Addition of Testosterone (short)	37.1 ± 3.3	0.74 ± 0.41	N/D	0.9 ± 0.7
	Addition of Testosterone (long)	36.9 ± 2.5	0.60 ± 0.32	N/D	0.9 ± 0.6
Tosun, 2021	Control	34.4 ± 2.6	0.3 ± 0.2	5.5 ± 1.4	N/D
	Addition of rLH	35.6 ± 4.6	0.4 ± 0.3	4.6 ± 1.5	N/D
Wang, 2022	Control	39.5 ± 4.4	0.49 ± 0.11	4.9 ± 2.8	N/D
	Addition of DHEA	39.0 ± 4.6	0.55 ± 0.10	5.1 ± 2.2	N/D
Yeung, 2015	Control	37.0 ± 0.8	0.64 ± 0.17	3.0 ± 0.3	N/D
	Addition of DHEA	36.0 ± 0.8	0.75 ± 0.19	4.0 ± 0.3	N/D
Younis, 2016	Control	38.6 ± 3.7	N/D	6.6 ± 2.5	N/D
	Addition of rLH	38.9 ± 2.8	N/D	6.0 ± 3.2	N/D
Zhang, 2014	Control	37.4 ± 4.3	1.12 ± 0.84	3.0 ± 1.4	N/D
	Addition of DHEA	37.2 ± 5.2	1.01 ± 0.77	2.9 ± 1.4	N/D
Zhang, 2022	Control	40.2 ± 3.7	1.00 ± 0.80	5.7 ± 3.0	N/D
	Addition of Estrogen	40.4 ± 4.1	1.00 ± 0.80	5.4 ± 2.9	N/D

*POR* poor ovarian response, *BMI* body mass index, *POR* poor ovarian response, *rLH* recombinant luteinizing hormone, *DHEA* Dehydroepiandrosterone, *AMH* anti-Müllerian hormone, *IU* international unit

Among the 22 included studies, all were RCTs [12, 13, 16, 17, 89–106]. Of these, 5 studies [13, 92, 95, 103, 105] where from China, 3 studies [89, 97, 99] were from Iran, 2 studies [16, 102] were from Turkey, 2 studies were from Greece [91, 100], 2 studies were from Egypt [90, 96], 1 study was from Denmark [94], 1 study was from Italy [98], 1 study was from Spain [101], 1 study was from Switzerland [17], 1 study was from Australia [12], 1 study was from Hong Kong [106], 1 study was from Israel [104], and 1 study was from Vietnam [93]. A total of 4131 infertile patients with a diagnosis of POR according to Bologna criteria [23] were included in this analysis; of these, 2069 patients underwent OS without hormonal add-ons and 2062 patients received hormonal additions in the context of OS. Two hundred ten patients were given LTZ as a hormonal add-on during the OS [16, 95, 97], 214 patients were given CC during the OS [17, 98, 100], 509 patients were given rLH during the OS [94, 102, 104], 276 patients were pre-treated with estrogen before the OS [13], 493 patients were pre-treated with DHEA before the OS [92, 103, 105, 106], 167 patients were pre-treated with testosterone before the OS [89, 91, 93, 99, 101], and 200 patients were given GH during the OS [12, 90, 96].

### Risk of bias of included studies

Figure S1a shows the quality of the methodology used in the 22 included RCTs, and Figure S1b shows a percentage-based assessment of the methodological quality of these trials. Overall, one RCT [90] was deemed at high risk of bias, six RCTs [89, 93, 98–101] were judged as having “some concerns” and 15 RCTs [12, 13, 16, 17, 92, 94–97, 102–106] were considered at low risk, respectively.

In detail, nine trials [90–92, 95, 98–100, 105, 106] had some or severe concerns for biases arising from the randomization process (Figure S1a-b). No study was deemed at high risk for bias due to deviations from intended interventions or missing data. On the contrary, there was a high risk of bias in the measurement of the outcomes in three studies [89, 93, 101]. Conversely, the vast majority of RCTs were at low risk for selection biases (Figure S1a-b).

All the included RCTs [12, 13, 16, 17, 70, 89–105] were registered in valid prospective repositories prior to the enrollment of the first woman.

Certainty of evidence according to CINeMA criteria ranged from very low to low, principally due to major concerns in the imprecision and incoherence domains, which were related to the low number of direct comparisons available (Supplementary Table 2). Potential publication bias was visually displayed in Figure S2a-g.

### Synthesis of results

#### Live birth rate

**Fig. 2:**
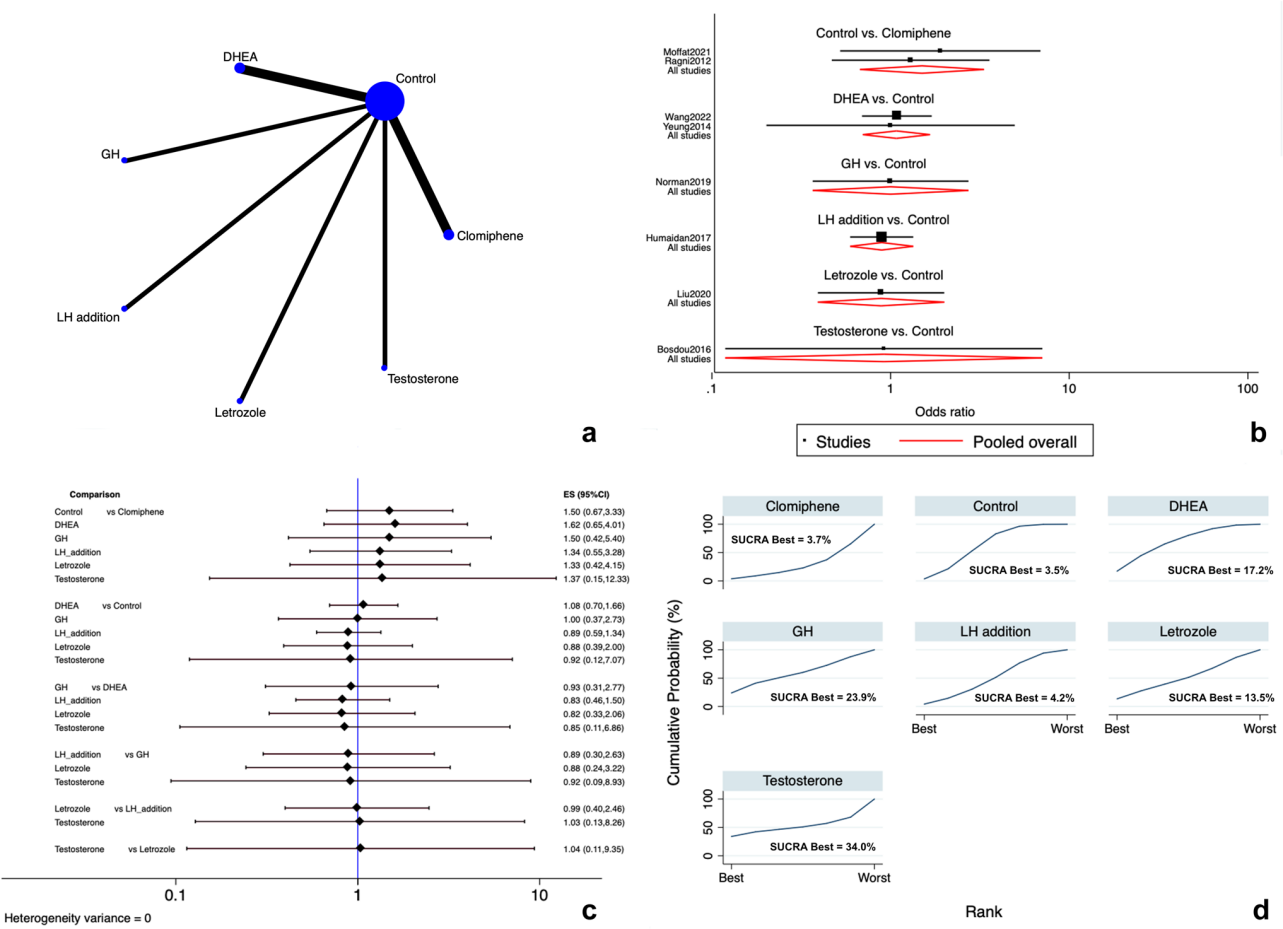
Live birth rate. **a** Network of comparisons of interventions analyzed in included studies. **b** Forest plot for the outcome. **c** Interval plot. **d** Ranking plot according to SUCRA analysis

Eight studies [12, 17, 91, 94, 95, 98, 103, 106] reported on the LBR. The following add-ons were directly compared with controls and indirectly compared among them: LTZ, CC, rLH, DHEA, testosterone, and GH. The network map for the outcome is shown in Fig. 2a**.** The general analysis for inconsistency revealed no degrees of inconsistency. As outlined in the forest and interval plots (Fig. 2b, c), there were no significant differences among all the evaluated add-ons (very low evidence) (Supplementary Table 2).

According to SUCRA ranking of available choices, testosterone (SUCRA = 34.0%) and GH (SUCRA = 23.9%) had the highest probability of being ranked treatment of choice (Fig. 2d). Publication bias for the LBR was not assessed since less than 10 studies reported the outcome.

#### Clinical pregnancy rate

**Fig. 3:**
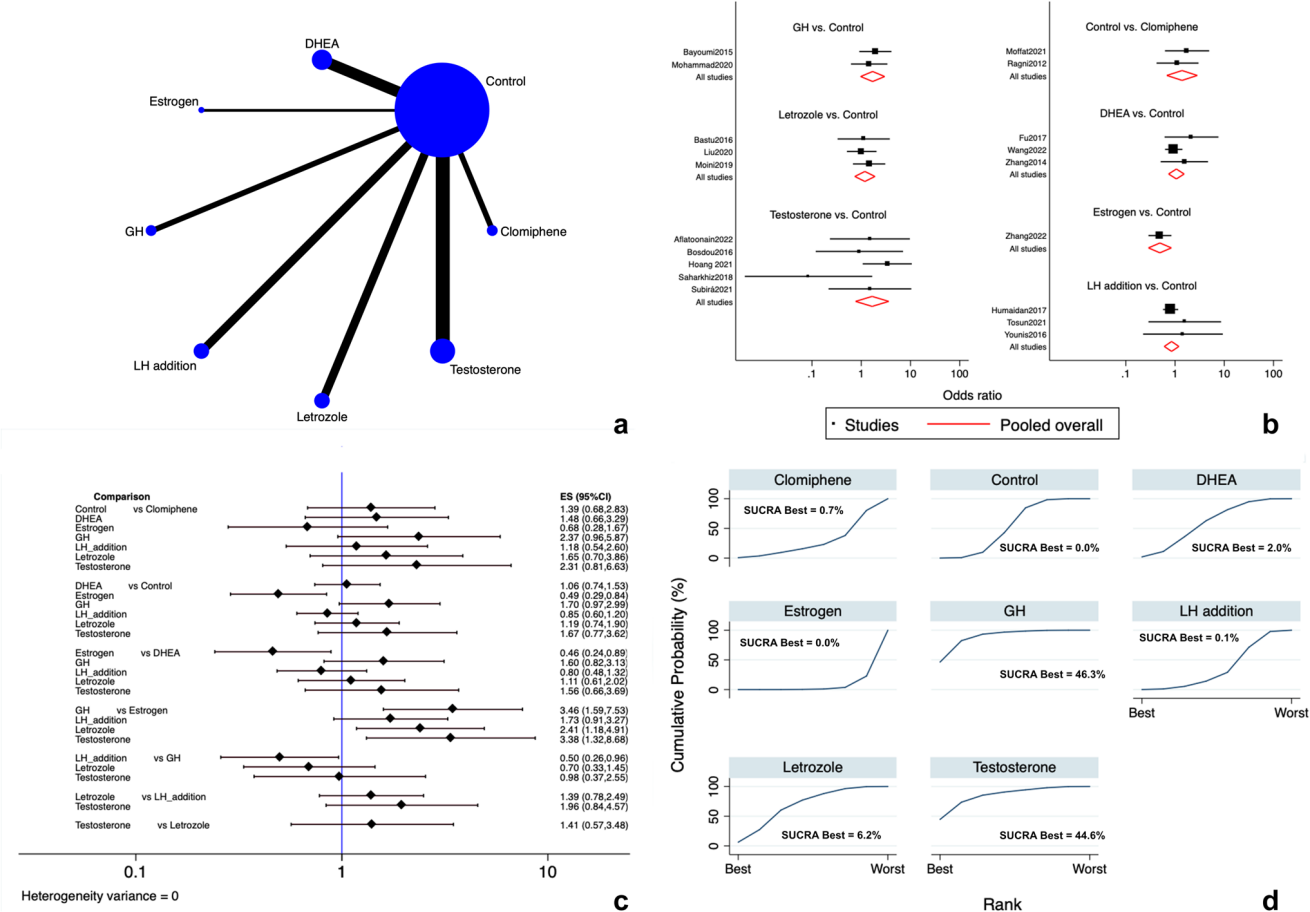
Clinical pregnancy rate. **a** Network of comparisons of interventions analyzed in included studies. **b** Forest plot for the outcome. **c** Interval plot. **d** Ranking plot according to SUCRA analysis

Nineteen RCTs [13, 16, 17, 89–99, 101–105] estimated the CPR. All the included interventions (LTZ, CC, rLH, estrogen, DHEA, testosterone and GH) were directly and indirectly compared. The network of direct comparisons is shown in Fig. 3a. No source of inconsistency was notable. The forest and interval plots displayed in Fig. 3b and c show the results of direct and indirect comparisons for CPR. GH was more efficacious than adding estrogens (OR 3.46 [95% CI 1.59 to 7.53]; low evidence) while adding estrogens was less efficacious than controls (OR 0.49 [95% CI 0.29 to 0.84]; low evidence) and DHEA (OR 0.46 [95% CI 0.24 to 0.89]; very low evidence). Similarly, adding rLH was significantly less efficacious than GH (OR 0.50 [95% CI 0.26 to 0.96]; very low evidence). No significant differences were retrieved among the protocol comparisons (low to very low evidence) (Supplementary Table 2) (Fig. 3c). According to the SUCRA ranking, GH (SUCRA = 46.3%) and testosterone (SUCRA = 44.6%) had increased chances of being ranked first among the hormonal add-ons (Fig. 3d).Publication bias was not apparent in both Egger’s test (*p* = 0.329) and funnel plot analysis (Figure S2a).

#### Number of overall oocytes and MII oocytes retrieved

**Fig. 4:**
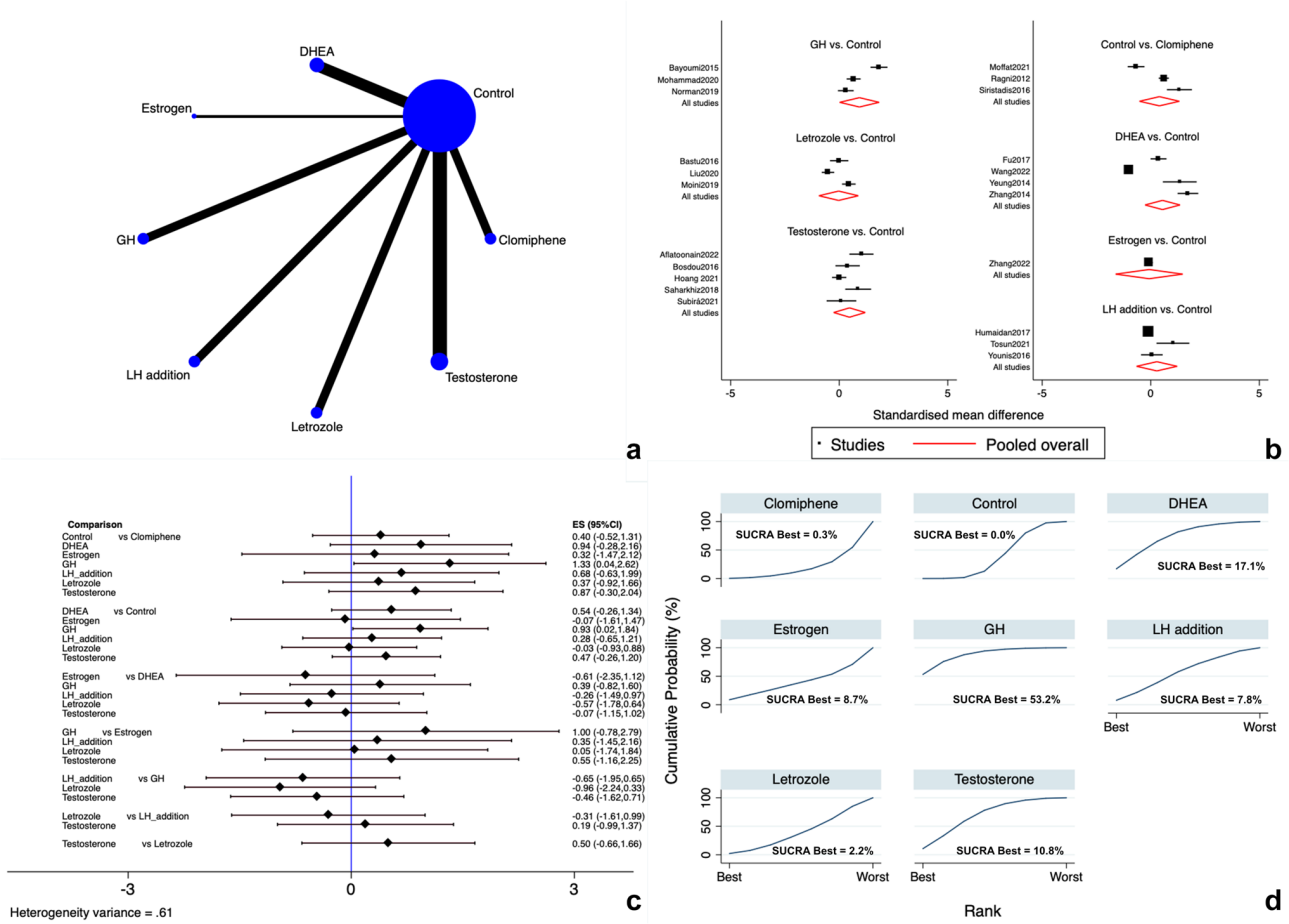
Mean number of retrieved oocytes. **a** Network of comparisons of interventions analyzed in included studies. **b** Forest plot for the outcome. **c** Interval plot. **d** Ranking plot according to SUCRA analysis

**Fig. 5:**
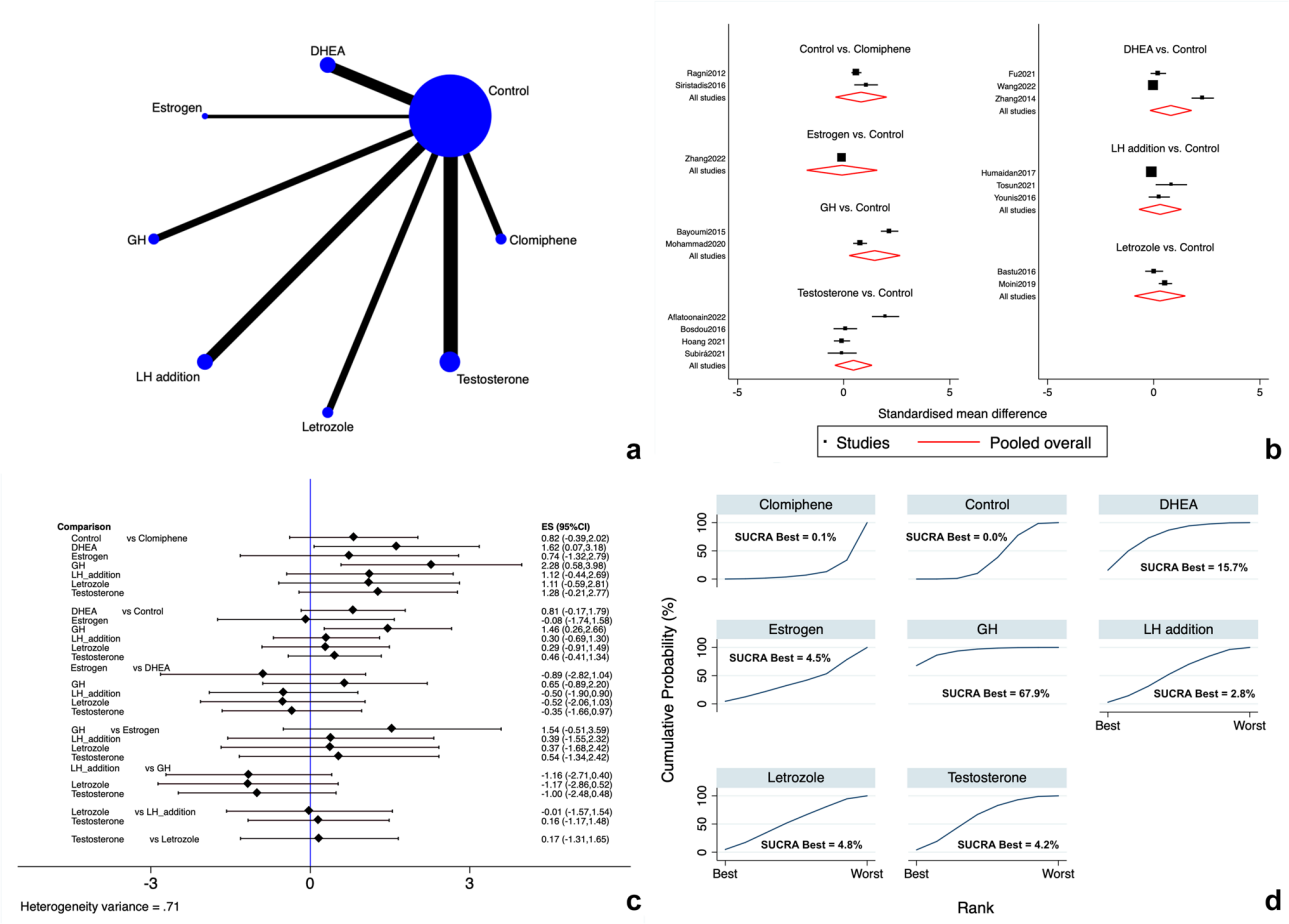
Mean number of M2 oocytes. **a** Network of comparisons of interventions analyzed in included studies. **b** Forest plot for the outcome. **c** Interval plot. **d** Ranking plot according to SUCRA analysis

Regarding the overall mean number of retrieved oocytes, reported in all the 22 studies [12, 13, 16, 17, 89–106], all the seven hormonal add-ons (LTZ, CC, rLH, estrogen, DHEA, testosterone, and GH) were directly and indirectly compared (Fig. 4a) without the presence of inconsistency in the general analysis. Forest and interval plots showed that GH was significantly more efficacious than CC (SMD 1.33 [95% CI 0.04 to 2.62]; very low evidence) and controls (SMD 0.93 [95% CI 0.02 to 1.84]; very low evidence) (Fig. 4b, c) with no other significant differences noted (very low evidence) (Supplementary Table 2). SUCRA analysis revealed that GH was likely the treatment of choice for gaining a higher number of retrieved oocytes (SUCRA = 53.2%) (Fig. 4 d).Egger’s test (*p* = 0.010) and funnel plot analysis (Figure S2e) reported the plausible presence of publication bias, warranting caution in the interpretation of the outcome.The mean number of MII oocytes was available in 17 trials [13, 16, 89–94, 96–98, 100–105] in which all the available hormonal add-ons (LTZ, CC, rLH, estrogen, DHEA, testosterone and GH) were compared (Fig. 5a). There were no degrees of inconsistency to report. Among all the add-on strategies, adding GH was more efficacious than CC (SMD 2.28 [95% CI 0.58 to 3.98]; very low evidence) and controls (SMD 1.46 [95% CI 0.26 to 2.66]; very low evidence). No other relevant differences were notable (very low evidence) (Supplementary Table 2) (Fig. 5b, c). According to the SUCRA ranking plot, GH was the add-on with the highest degree of best treatment (SUCRA = 67.9%) (Fig. 5 d).Both Egger’s test (*p* = 0.110) and funnel plot analysis (Figure S2f) reported no apparent publication bias.

#### Fertilization rate

The fertilization rate was reported in 14 studies [13, 16, 70, 89–94, 96–98, 100–105] comparing all the hormonal add-ons (LTZ, CC, rLH, estrogen, DHEA, testosterone, and GH) eligible for this network meta-analysis (Figure S3a). No degrees of inconsistency were retrieved in the general analysis. As shown in forest and interval plots (Figure S3b-c), adding estrogens was significantly less efficacious than the control group (OR 0.49 [95% CI 0.29 to 0.84]; low evidence) while GH was significantly more efficacious than estrogen too (OR 3.14 [95% CI 1.50 to 6.57]; low evidence). There were no other significant differences between treatments and controls (very low evidence) (Supplementary Table 2) (Fig. S3c). SUCRA analysis revealed that rLH addition (SUCRA = 37.3%), DHEA (SUCRA = 18.8%) and testosterone (SUCRA = 14.8%) had the highest likelihood of being treatments of choice for raising the fertilization rate (Figure S3d).According to Egger’s test (*p* = 0.376) and funnel plot analysis (Figure S2g), there were no apparent issues of publication bias.

#### Duration of OS

Fifteen studies [12, 13, 16, 17, 90, 91, 93, 95–97, 100–103, 106], directly and indirectly comparing all the add-ons, reported the outcome (Figure S4a). According to forest and interval plots (Figure S4b-c), there were no significant differences among the approaches (very low evidence) (Supplementary Table 2).SUCRA analysis reported that LTZ had increased chances of being the add-on with the shortest OS duration (SUCRA = 52.0%) (Figure S4d). No publication bias was reported in Egger’s test (*p* = 0.423) and funnel plot analysis (Figure S2b).

#### Total dose of gonadotropins

Out of 15 studies [12, 13, 16, 17, 70, 90, 91, 93–95, 97, 100, 102, 103, 106] reporting the mean total dose of gonadotropins used during COS for all the hormonal add-ons (LTZ, CC, rLH, estrogen, DHEA, testosterone and GH) (Figure S5a), no significant differences were notable (low to very low evidence) (Supplementary Table 2) among treatments except for a significantly smaller number of used gonadotropins with LTZ relative to controls (SMD –7.02 [95% CI − 12.82 to − 1.22]; low evidence) (Figures S5b-c).According to the SUCRA ranking plot, LTZ (SUCRA = 67.0%) had the greatest degree of best treatment for the outcome (Figure S5d).

Publication bias was not apparent in both Egger’s test (*p* = 0.339) and funnel plot analysis (Figure S2c).

#### Estrogen levels at trigger day

The outcome was reported in 14 studies [13, 17, 89–93, 95–97, 102–104, 106] in which all the hormone add-ons (LTZ, CC, rLH, estrogen, DHEA, testosterone, and GH) were compared (Figure S6a). The consistency analysis showed no source of inconsistency. Figures S6b and S6c show the forest and interval plots, in which estrogen levels were significantly less with the use of LTZ relative to CC (SMD − 3.47 [95% CI − 6.85 to − 0.01]; very low evidence), controls (SMD − 2.42 [95% CI − 4.38 to − 0.47]; very low evidence) and GH (SMD − 4.04 [95% CI − 6.80 to − 1.28]; very low evidence). Similarly, testosterone was more efficacious than LTZ (SMD 2.83 [95% CI 0.31 to 5.36]; very low evidence). No other differences were notable (very low evidence) (Supplementary Table 2) (Figure S6c). According to SUCRA analysis, GH (SUCRA = 46.3%) was the add-on with the highest likelihood of best treatment (Figure S6d).

According to Egger’s test (*p* = 0.913) and funnel plot analysis (Figure S2d), no publication bias was apparent.

#### Cancellation rate

With all the evaluated hormonal add-ons (LTZ, CC, rLH, estrogen, DHEA, testosterone, and GH), eight RCTs [13, 16, 91, 94–96, 98, 101] reported the cancellation rate (Figure S7a). There were zero degrees of inconsistency to report.

According to forest and interval plots (Figure S7b-c), no significant differences among all the add-ons were notable (very low evidence) (Supplementary Table 2). SUCRA analysis revealed that GH (SUCRA = 45.1%) was the most likely best-ranked treatment (Figure S7d). Publication bias was not evaluated since less than 10 studies reported the outcome.

## Discussion

### Principal findings

The aim of this systematic review and network meta-analysis of RCTs was to evaluate the reproductive outcomes of hormonal add-ons in women with POR undergoing ART. For the LBR, which was our primary outcome, no significant differences were observed between the evaluated interventions; however, testosterone showed the highest probability of improving LBR according to SUCRA analysis. Meanwhile, GH showed the highest SUCRA score for CPR. Moreover, GH also demonstrated better results in terms of total number of retrieved oocytes and mean number of MII oocytes. Finally, LTZ demonstrated benefits in reducing gonadotropin usage and stimulation duration.

### Comparison with existing literature

Several add-ons to OS have been explored in POR women. However, available studies did not ensure their effectiveness. A recent meta-analysis of RCTs concluded that pretreatment with testosterone appears to be related to a better LBR in women with POR [21]. The authors of the study, however, compared not only hormonal add-ons with controls, but also variations in OS protocols including GnRH agonists or antagonists, different types of gonadotropins, and dual-stimulation regimen. This heterogeneity, combined with the limitations derived from a classic meta-analysis approach, makes it difficult to interpret how their conclusions were derived. In fact, a conventional pairwise meta-analysis, which only compares treatments directly from primary studies, does not allow treatments to be ranked according to their efficacy or safety, offering a more limited basis for clinical decision-making. Because the ideal goal should be to compare available hormonal add-on therapies within each other to achieve a broader and more quantitative view of their comparative effectiveness of, a network meta-analytic approach should be preferred, as performed by Zhang et al. in 2020 [22]. However, this study is not without strong limitations. First, the reliability of the included studies has not been evaluated and is not adequate to the actual trustworthiness standards [107]. In addition, although the authors claimed to include only patients defined as POR according to the March 2010 Bologna criteria of the European Society of Human Reproduction and Embryology (ESHRE) [23], multiple studies published before this date were included, some of which enrolled patients who could not be defined as POR according to them [23]. This lower homogeneity of the included population consequently significantly reduces the clinical and methodological variability and thus also the reliability and generalizability of the results. Moreover, a crucial strength of our analysis compared to that of Zhang et al. [22] concerns the primary outcome analyzed. The choice of LBR as the primary outcome, compared to the CPR used by the authors of the previous network meta-analysis, represents a substantial advantage. In fact, the ESHRE explicitly recommends the use of LBR as the primary outcome in clinical trials and systematic reviews conducted with the aim to evaluate the clinical effectiveness of using add-ons in ART treatments, as LBR is considered the most clinically relevant parameter, being directly related to the final success of treatment and reflecting the ultimate goal of couples undertaking reproductive medicine treatment [108]. Therefore, the use of LBR as the primary endpoint in this network meta-analysis significantly increases its clinical relevance and relevance to the information needs of physicians and patients, clearly distinguishing it from the previous meta-analysis by Zhang et al. [22].

It is crucial to underline that SUCRA rankings reflect the probability that an intervention is among the best options based on the entire network of evidence. However, a higher SUCRA score does not imply that the intervention is statistically superior to others in direct or indirect comparisons. In our analysis, no statistically significant differences were observed among most pairwise comparisons, which suggests limited certainty in the relative superiority of the treatments. Nevertheless, the consistency of higher SUCRA values for GH and testosterone across multiple outcomes suggests a favorable trend that warrants further investigation. Therefore, SUCRA rankings should be interpreted as hypothesis-generating tools rather than definitive clinical guidance, particularly in this scenario where statistical significance is lacking. Our findings highlight GH and testosterone as the agents with the highest probability of improving reproductive outcomes in women with POR undergoing ART.

The exact mechanism by which GH might improve the outcomes of OS remains unclear. However, it is known that GH enhances insulin-like growth factor 1 (IGF-1) activity in the follicle, which plays a critical role in granulosa cell function and follicular maturation [109]. The studies included in our meta-analysis [12, 18, 90] reported a higher number of oocytes retrieved after the administration of GH in POR women. Nevertheless, the benefit for the LBR could not be clearly demonstrated. A review by Kolibianakis et al. in 2009 [110] had already suggested a significant increase in the number of retrieved oocytes and an improvement in LBR when GH was added to OS in poor responders. However, with the implementation of Bologna criteria, recent reviews concluded that the effect of GH administration on ovarian response or pregnancy rate is still contradictory and remains unclear [21, 111]. A recent Cochrane on this topic also reported similar results [112]. Conversely, the use of adjuvant GH in OS protocols has an uncertain effect on LBR and the average number of oocytes retrieved in patients expected to be good responders. Therefore, although promising, the use of GH as an add-on in women with POR requires further high-quality studies to confirm its efficacy, given the paucity of trials and their inherent biases and imprecisions.

Androgens play a crucial role in folliculogenesis. Specifically, they influence the proliferation of granulosa cells, the number of growing follicles as well as the expression of FSH receptors in the ovary [113]. Different agents have been explored to increase intraovarian androgen concentration: transdermal testosterone, DHEA, and androgen modulators such as LTZ. Our findings suggest that transdermal testosterone could have a positive impact on the LBR and CPR. However, the available evidence regarding the clinical utility of transdermal testosterone in poor responders shows contradictory results. Some authors reported an increase in pregnancy rates [93, 99] and ovarian response [89, 99] after testosterone administration. In contrast, well-designed clinical trials did not show differences in their primary outcomes (number of oocytes and pregnancy rate) using testosterone [91, 101]. Recently, a conference abstract of a multicenter multinational RCT concluded that testosterone pre-treatment prior to IVF/ICSI does not increase clinical pregnancy rates in poor ovarian responders [114]. Nevertheless, a recent meta-analysis [115] concluded that the probability of obtaining more oocytes and clinical pregnancies was significantly increased in women pretreated with transdermal testosterone compared with those who were not, with a lower cancellation rate in the testosterone group. However, available studies lack homogeneity regarding type, dose, and duration of testosterone supplementation. Thus, although it is suggested that the use of testosterone in POR women may improve reproductive outcomes, a consensus on the supplementation protocol needs to be reached.

In the current meta-analysis, the use of DHEA has shown no benefit in improving reproductive outcomes, except for a decrease in the cancellation rate. This contrasts with other reviews and meta-analyses suggesting that DHEA could improve oocyte response and pregnancy rates in patients with poor response [21, 22].

Another add-on that showed some results in our network meta-analysis was LTZ. LTZ is an aromatase inhibitor that increases intraovarian androgens by inhibiting the conversion of androgens into estrogens. In concordance with other authors [21], we did not observe a benefit with LTZ in the number of oocytes retrieved or pregnancy rate although we observed a reduction of gonadotropin consumption and duration of stimulation with the use of LTZ. Only a few studies have evaluated the use of this add-on in patients with POR [8, 16, 97]. Authors who have evaluated the use of LTZ in mild stimulation compared to conventional stimulation without LTZ have observed no differences in reproductive outcomes [8, 16]. However, Moini et al. [97] showed that patients treated with conventional doses of gonadotrophins plus LTZ had an increase in the number of oocytes retrieved along with a decrease in gonadotropin consumption and duration of stimulation compared to the women without LTZ addition. LTZ is therefore a promising treatment that requires further exploration. According to our results, neither the use of CC, estrogen priming nor the addition of rLH would gain an additional benefit in patients with POR.

While this network meta-analysis was limited to hormonal add-ons, other interventions such as coenzyme Q10, low-dose hCG, and corifollitropin alfa showed promising potential in improving ovarian response. Coenzyme Q10, through its antioxidant and mitochondrial-supportive properties, may enhance oocyte quality in women with POR [19]. Low-dose hCG, when added during OS, may mimic LH activity and support follicular maturation, although high-quality RCTs in Bologna-defined POR are lacking [116]. Corifollitropin alfa offers the advantage of simplified stimulation protocols with a single injection replacing daily FSH, and may be useful in selected POR [117]. However, the current body of evidence is either methodologically limited or inconsistent with our inclusion criteria. Future high-quality RCTs are needed to assess the role of such agents and determine their integration into individualized OS strategies.

### Strengths and limitations

Several limitations should be considered while interpreting our findings. The certainty of evidence was primarily low due to imprecision and incoherence, mainly stemming from the limited number of direct comparisons available and variations in study methodologies.

The dosages, the beginning time, the length of ovulation stimulation, and the GnRH analog protocols employed in the suggested interventions and OS protocols also showed notable differences. Therefore, some small heterogeneity among included trials may have influenced the findings. Due to the small sample sizes of the eligible trials, it was typically impossible to properly investigate this clinical heterogeneity. Consequently, the current systematic review and network meta-analysis permitted only broad comparisons between various hormonal adjuvant treatments and no treatment, lowering the certainty of evidence for imprecision due to the lack of significant differences in several comparisons. Future research will allow for the assessment of a possible moderating influence of these parameters on the effectiveness of the interventions under investigation.

Conversely, to ensure the robustness and validity of such findings, we included only studies in which the POR followed the Bologna criteria [23], excluding many trials that might increase inherent heterogeneity and thus be a strength of this network meta-analysis.

Moreover, all the most critical ART outcomes, including also the LBR, were analyzed, emphasizing the importance of our findings in clinical practice. Additionally, the consistency of the reported findings, showing agreement in the top-ranked add-ons (GH and testosterone) for most study outcomes, provides useful indications for routine practice in practitioners dealing with women with POR undergoing ART.

In conclusion, the results of the present systematic review and network meta-analysis of RCTs suggest that the addition of testosterone and GH may offer benefits to POR patients undergoing OS. The addition of LTZ also showed some sort of benefit, showing the improvement of some OS parameters. In contrast, the addition of DHEA, CC, estrogen, or rLH did not show significant benefit. However, the current certainty of evidence is still limited to low and very low; therefore, it is crucial to further investigate the real clinical value of these additional hormone treatments with further rigorous studies.

## Supplementary Information

Below is the link to the electronic supplementary material.

Supplementary file1 (DOCX 115 KB)

Supplementary file2 (DOCX 151 KB)

(PNG 621 KB)

(PNG 83.9 KB)

(PNG 481 KB)

(PNG 376 KB)

(PNG 362 KB)

(PNG 376 KB)

(PNG 356 KB)

(PNG 213 KB)

## Data Availability

Raw data for this network meta-analysis are available at the corresponding author upon reasonable request.

## Declarations

### Attestation statement

• All the data analyzed in this systematic review and meta-analysis has been published in the evaluated trials.

• Data will be made available to the editors of the journal for review or query upon.

request.
