# Supplementary material for: Effectiveness of hormone add-on strategies in ovarian stimulation for women with poor ovarian response: a systematic review and network meta-analysis of randomized controlled trials
Source: J Assist Reprod Genet. 2025 Oct 25;42(10):3231–52. doi: 10.1007/s10815-025-03633-z (PMC12602825; doi:10.1007/s10815-025-03633-z)
Supplement: Supplementary file 1 — Supplementary file1 (DOCX 115 KB) [file 10815_2025_3633_MOESM1_ESM.docx]

**Supplementary Table 1.** Excluded full-texts with reason for exclusion

| Study, year | Reason for exclusion | Reference |
| --- | --- | --- |
| Schoolcraft, 2008 | No RCT |  |
| Bulow, 2022 | No Bologna POR |  |
| yang, 2019 | No Bologna POR |  |
| Fouda, 2011 | Inappropriate controls |  |
| Bulow, 2023 | No Bologna POR |  |
| Abdel Mohsen, 2013 | No trial registration |  |
| Ozmen, 2009 | No trial registration |  |
| Davar, 2009 | No trial registration |  |
| Nabati, 2016 | Registered after start |  |
| Ebrahimi, 2017 | Registered after start |  |
| Revelli 2014 | No trial registration |  |
| Kara, 2013 | No Bologna POR |  |
| Kim, 2011 | No Bologna POR |  |
| Elprince, 2020 | Retracted |  |
| Choe, 2017 | No trial registration |  |
| Eftekhar, 2012 | No trial registration |  |
| Lee, 2019 | No RCT |  |
| Mohammadi, 2021 | No Hormonal add-on |  |
| Xu, 2018 | No Hormonal add-on |  |
| Song, 2021 | No Hormonal add-on |  |
| Jinno, 2023 | No Hormonal add-on |  |
| Barrenetxea, 2008 | No Bologna POR |  |
| Berkkanoglu, 2007 | Inappropriate controls |  |
| Musters, 2011 | No trial registration |  |
| Ferraretti, 2014 | No trial registration |  |
| Parinaud, 1992 | Inappropriate controls |  |
| Mak, 2017 | Inappropriate controls |  |
| Revelli, 2012 | Inappropriate controls |  |
| De Placido, 2006 | Inappropriate controls |  |
| Lok, 2004 | No Hormonal add-on |  |
| Kalra, 2008 | No Bologna POR |  |
| Kucuk, 2007 | No Bologna POR |  |
| Kucuk, 2008 | No Bologna POR |  |
| Yeung, 2015 | No Bologna POR |  |
| Llácer, 2020 | No Hormonal add-on |  |
| Maged, 2015 | No Hormonal add-on |  |
| Esfidani, 2021 | No Hormonal add-on |  |
| Aflatoonian, 2017 | No Hormonal add-on |  |
| Ashrafi, 2018 | No Hormonal add-on |  |
| Zarei, 2018 | No Hormonal add-on |  |
| Yu, 2018 | No Hormonal add-on |  |
| Ghaffari, 2019 | No Hormonal add-on |  |
| Malmusi, 2005 | No Hormonal add-on |  |
| Merviel, 2015 | No Hormonal add-on |  |
| Lainas, 2008 | No Hormonal add-on |  |
| Demirol, 2009 | No Hormonal add-on |  |
| DiLuigi, 2011 | No Hormonal add-on |  |
| Kahraman, 2009 | No Hormonal add-on |  |
| Karimzadeh, 2010 | No Hormonal add-on |  |
| Davar, 2010 | No Hormonal add-on |  |
| Cheung, 2005 | No Hormonal add-on |  |
| Prapas, 2012 | No Hormonal add-on |  |
| Youssef, 2017 | No Hormonal add-on |  |
| Marci, 2005 | No Hormonal add-on |  |
| Tazegül, 2008 | No Hormonal add-on |  |
| Sunkara, 2014 | No Hormonal add-on |  |
| Weissman, 2003 | No Hormonal add-on |  |
| Chen, 2019 | No Hormonal add-on |  |
| Kotb2016 | Untrustworthy |  |
| Pilehvari, 2016 | Registered after start |  |
| Revelli, 2020 | Inappropriate controls |  |
| F.Raga, 1999 | Inappropriate controls |  |
| A.Akman, 2000 | No Bologna POR |  |
| A.Akman, 2001 | No Bologna POR |  |
| Berkkanoglu, 2010 | Inappropriate controls |  |
| Cedrin-Durnerin, 1999 | Inappropriate controls |  |
| Lefebvre, 2015 | Inappropriate controls |  |
| Cerrillo, 2023 | No Hormonal add-on |  |
| Massin, 2023 | No Hormonal add-on |  |
| Kolibianakis, 2015 | No Hormonal add-on |  |
| Drakopoulos, 2017 | No Hormonal add-on |  |
| Fusi, 2020 | No Hormonal add-on |  |
| Liu, 2023 | No Hormonal add-on |  |
| Van Tilborg, 2017 | No Hormonal add-on |  |
| Klinkert, 2005 | No Hormonal add-on |  |
| Dakhly2018 | Expression of concern |  |
| Kucuk, 2008 -2 | No Bologna POR |  |
