# Supplementary material for: Effectiveness of hormone add-on strategies in ovarian stimulation for women with poor ovarian response: a systematic review and network meta-analysis of randomized controlled trials
Source: J Assist Reprod Genet. 2025 Oct 25;42(10):3231–52. doi: 10.1007/s10815-025-03633-z (PMC12602825; doi:10.1007/s10815-025-03633-z)
Supplement: Supplementary file 2 — Supplementary file2 (DOCX 151 KB) [file 10815_2025_3633_MOESM2_ESM.docx]

**Supplementary Table 1**. Certainty of evidence according to CiNEMA criteria.

Live birth rate

| **Comparison** | **Number of studies** | **Within-study bias** | **Reporting bias** | **Indirectness** | **Imprecision** | **Heterogeneity** | **Incoherence** | **Confidence rating** |
| --- | --- | --- | --- | --- | --- | --- | --- | --- |
| **Clomiphene:Control** | 2 | Some concerns | Low risk | No concerns | Major concerns | No concerns | Major concerns | **Very low** |
| **Control:DHEA** | 2 | No concerns | Low risk | No concerns | Major concerns | No concerns | Major concerns | **Very low** |
| **Control:GH** | 1 | No concerns | Low risk | No concerns | Major concerns | No concerns | Major concerns | **Very low** |
| **Control:LH addition** | 1 | No concerns | Low risk | No concerns | Major concerns | No concerns | Major concerns | **Very low** |
| **Control:Letrozole** | 1 | No concerns | Low risk | No concerns | Major concerns | No concerns | Major concerns | **Very low** |
| **Control:Testosterone** | 1 | No concerns | Low risk | No concerns | Major concerns | No concerns | Major concerns | **Very low** |
| **Clomiphene:DHEA** | 0 | No concerns | Low risk | No concerns | Major concerns | No concerns | Major concerns | **Very low** |
| **Clomiphene:GH** | 0 | No concerns | Low risk | No concerns | Major concerns | No concerns | Major concerns | **Very low** |
| **Clomiphene:LH addition** | 0 | No concerns | Low risk | No concerns | Major concerns | No concerns | Major concerns | **Very low** |
| **Clomiphene:Letrozole** | 0 | No concerns | Low risk | No concerns | Major concerns | No concerns | Major concerns | **Very low** |
| **Clomiphene:Testosterone** | 0 | No concerns | Low risk | No concerns | Major concerns | No concerns | Major concerns | **Very low** |
| **DHEA:GH** | 0 | No concerns | Low risk | No concerns | Major concerns | No concerns | Major concerns | **Very low** |
| **DHEA:LH addition** | 0 | No concerns | Low risk | No concerns | Major concerns | No concerns | Major concerns | **Very low** |
| **DHEA:Letrozole** | 0 | No concerns | Low risk | No concerns | Major concerns | No concerns | Major concerns | **Very low** |
| **DHEA:Testosterone** | 0 | No concerns | Low risk | No concerns | Major concerns | No concerns | Major concerns | **Very low** |
| **GH:LH addition** | 0 | No concerns | Low risk | No concerns | Major concerns | No concerns | Major concerns | **Very low** |
| **GH:Letrozole** | 0 | No concerns | Low risk | No concerns | Major concerns | No concerns | Major concerns | **Very low** |
| **GH:Testosterone** | 0 | No concerns | Low risk | No concerns | Major concerns | No concerns | Major concerns | **Very low** |
| **Letrozole:LH addition** | 0 | No concerns | Low risk | No concerns | Major concerns | No concerns | Major concerns | **Very low** |
| **LH addition:Testosterone** | 0 | No concerns | Low risk | No concerns | Major concerns | No concerns | Major concerns | **Very low** |
| **Letrozole:Testosterone** | 0 | No concerns | Low risk | No concerns | Major concerns | No concerns | Major concerns | **Very low** |

Clinical pregnancy rate

| **Comparison** | **Number of studies** | **Within-study bias** | **Reporting bias** | **Indirectness** | **Imprecision** | **Heterogeneity** | **Incoherence** | **Confidence rating** |
| --- | --- | --- | --- | --- | --- | --- | --- | --- |
| **Clomiphene:Control** | 2 | Some concerns | Low risk | No concerns | Major concerns | No concerns | Major concerns | **Very low** |
| **Control:DHEA** | 4 | No concerns | Low risk | No concerns | Major concerns | No concerns | Major concerns | **Very low** |
| **Control:Estrogen** | 1 | No concerns | Low risk | No concerns | No concerns | No concerns | Major concerns | **Low** |
| **Control:GH** | 2 | Major concerns | Low risk | No concerns | Major concerns | No concerns | Major concerns | **Very low** |
| **Control:LH addition** | 2 | No concerns | Low risk | No concerns | Major concerns | No concerns | Major concerns | **Very low** |
| **Control:Letrozole** | 3 | No concerns | Low risk | No concerns | Major concerns | No concerns | Major concerns | **Very low** |
| **Control:Testosterone** | 5 | Some concerns | Low risk | No concerns | Major concerns | No concerns | Major concerns | **Very low** |
| **Clomiphene:DHEA** | 0 | No concerns | Low risk | No concerns | Major concerns | No concerns | Major concerns | **Very low** |
| **Clomiphene:Estrogen** | 0 | No concerns | Low risk | No concerns | Major concerns | No concerns | Major concerns | **Very low** |
| **Clomiphene:GH** | 0 | No concerns | Low risk | No concerns | Major concerns | No concerns | Major concerns | **Very low** |
| **Clomiphene:LH addition** | 0 | No concerns | Low risk | No concerns | Major concerns | No concerns | Major concerns | **Very low** |
| **Clomiphene:Letrozole** | 0 | No concerns | Low risk | No concerns | Major concerns | No concerns | Major concerns | **Very low** |
| **Clomiphene:Testosterone** | 0 | Some concerns | Low risk | No concerns | Major concerns | No concerns | Major concerns | **Very low** |
| **DHEA:Estrogen** | 0 | No concerns | Low risk | No concerns | No concerns | Major concerns | Major concerns | **Very low** |
| **DHEA:GH** | 0 | No concerns | Low risk | No concerns | Major concerns | No concerns | Major concerns | **Very low** |
| **DHEA:LH addition** | 0 | No concerns | Low risk | No concerns | Major concerns | No concerns | Major concerns | **Very low** |
| **DHEA:Letrozole** | 0 | No concerns | Low risk | No concerns | Major concerns | No concerns | Major concerns | **Very low** |
| **DHEA:Testosterone** | 0 | No concerns | Low risk | No concerns | Major concerns | No concerns | Major concerns | **Very low** |
| **Estrogen:GH** | 0 | No concerns | Low risk | No concerns | No concerns | No concerns | Major concerns | **Low** |
| **Estrogen:LH addition** | 0 | No concerns | Low risk | No concerns | Major concerns | No concerns | Major concerns | **Very low** |
| **Estrogen:Letrozole** | 0 | No concerns | Low risk | No concerns | No concerns | No concerns | Major concerns | **Low** |
| **Estrogen:Testosterone** | 0 | No concerns | Low risk | No concerns | No concerns | No concerns | Major concerns | **Low** |
| **GH:LH addition** | 0 | No concerns | Low risk | No concerns | No concerns | Major concerns | Major concerns | **Very low** |
| **GH:Letrozole** | 0 | No concerns | Low risk | No concerns | Major concerns | No concerns | Major concerns | **Very low** |
| **GH:Testosterone** | 0 | Some concerns | Low risk | No concerns | Major concerns | No concerns | Major concerns | **Very low** |
| **Letrozole:LH addition** | 0 | No concerns | Low risk | No concerns | Major concerns | No concerns | Major concerns | **Very low** |
| **LH addition:Testosterone** | 0 | No concerns | Low risk | No concerns | Major concerns | No concerns | Major concerns | **Very low** |
| **Letrozole:Testosterone** | 0 | No concerns | Low risk | No concerns | Major concerns | No concerns | Major concerns | **Very low** |

Fertilization rate

| **Comparison** | **Number of studies** | **Within-study bias** | **Reporting bias** | **Indirectness** | **Imprecision** | **Heterogeneity** | **Incoherence** | **Confidence rating** |
| --- | --- | --- | --- | --- | --- | --- | --- | --- |
| **Clomiphene:Control** | 2 | Some concerns | Low risk | No concerns | Major concerns | No concerns | Major concerns | **Very low** |
| **Control:DHEA** | 2 | No concerns | Low risk | No concerns | Major concerns | No concerns | Major concerns | **Very low** |
| **Control:Estrogen** | 1 | No concerns | Low risk | No concerns | No concerns | No concerns | Major concerns | **Low** |
| **Control:GH** | 3 | No concerns | Low risk | No concerns | Major concerns | No concerns | Major concerns | **Very low** |
| **Control:LH addition** | 1 | No concerns | Low risk | No concerns | Major concerns | No concerns | Major concerns | **Very low** |
| **Control:Letrozole** | 3 | No concerns | Low risk | No concerns | Major concerns | No concerns | Major concerns | **Very low** |
| **Control:Testosterone** | 1 | No concerns | Low risk | No concerns | Major concerns | No concerns | Major concerns | **Very low** |
| **Clomiphene:DHEA** | 0 | No concerns | Low risk | No concerns | Major concerns | No concerns | Major concerns | **Very low** |
| **Clomiphene:Estrogen** | 0 | No concerns | Low risk | No concerns | Major concerns | No concerns | Major concerns | **Very low** |
| **Clomiphene:GH** | 0 | Some concerns | Low risk | No concerns | Major concerns | No concerns | Major concerns | **Very low** |
| **Clomiphene:LH addition** | 0 | No concerns | Low risk | No concerns | Major concerns | No concerns | Major concerns | **Very low** |
| **Clomiphene:Letrozole** | 0 | No concerns | Low risk | No concerns | Major concerns | No concerns | Major concerns | **Very low** |
| **Clomiphene:Testosterone** | 0 | No concerns | Low risk | No concerns | Major concerns | No concerns | Major concerns | **Very low** |
| **DHEA:Estrogen** | 0 | No concerns | Low risk | No concerns | Major concerns | No concerns | Major concerns | **Very low** |
| **DHEA:GH** | 0 | No concerns | Low risk | No concerns | Major concerns | No concerns | Major concerns | **Very low** |
| **DHEA:LH addition** | 0 | No concerns | Low risk | No concerns | Major concerns | No concerns | Major concerns | **Very low** |
| **DHEA:Letrozole** | 0 | No concerns | Low risk | No concerns | Major concerns | No concerns | Major concerns | **Very low** |
| **DHEA:Testosterone** | 0 | No concerns | Low risk | No concerns | Major concerns | No concerns | Major concerns | **Very low** |
| **Estrogen:GH** | 0 | No concerns | Low risk | No concerns | No concerns | No concerns | Major concerns | **Low** |
| **Estrogen:LH addition** | 0 | No concerns | Low risk | No concerns | Major concerns | No concerns | Major concerns | **Very low** |
| **Estrogen:Letrozole** | 0 | No concerns | Low risk | No concerns | No concerns | Major concerns | Major concerns | **Very low** |
| **Estrogen:Testosterone** | 0 | No concerns | Low risk | No concerns | Major concerns | No concerns | Major concerns | **Very low** |
| **GH:LH addition** | 0 | No concerns | Low risk | No concerns | Major concerns | No concerns | Major concerns | **Very low** |
| **GH:Letrozole** | 0 | No concerns | Low risk | No concerns | Major concerns | No concerns | Major concerns | **Very low** |
| **GH:Testosterone** | 0 | No concerns | Low risk | No concerns | Major concerns | No concerns | Major concerns | **Very low** |
| **Letrozole:LH addition** | 0 | No concerns | Low risk | No concerns | Major concerns | No concerns | Major concerns | **Very low** |
| **LH addition:Testosterone** | 0 | No concerns | Low risk | No concerns | Major concerns | No concerns | Major concerns | **Very low** |
| **Letrozole:Testosterone** | 0 | No concerns | Low risk | No concerns | Major concerns | No concerns | Major concerns | **Very low** |

Cancellation rate

| **Comparison** | **Number of studies** | **Within-study bias** | **Reporting bias** | **Indirectness** | **Imprecision** | **Heterogeneity** | **Incoherence** | **Confidence rating** |
| --- | --- | --- | --- | --- | --- | --- | --- | --- |
| **Clomiphene:Control** | 1 | Some concerns | Low risk | No concerns | Major concerns | No concerns | Major concerns | **Very low** |
| **Control:DHEA** | 1 | No concerns | Low risk | No concerns | Major concerns | No concerns | Major concerns | **Very low** |
| **Control:Estrogen** | 1 | No concerns | Low risk | No concerns | Major concerns | No concerns | Major concerns | **Very low** |
| **Control:GH** | 1 | No concerns | Low risk | No concerns | Major concerns | No concerns | Major concerns | **Very low** |
| **Control:LH addition** | 1 | No concerns | Low risk | No concerns | Major concerns | No concerns | Major concerns | **Very low** |
| **Control:Letrozole** | 2 | No concerns | Low risk | No concerns | Major concerns | No concerns | Major concerns | **Very low** |
| **Control:Testosterone** | 2 | Some concerns | Low risk | No concerns | Major concerns | No concerns | Major concerns | **Very low** |
| **Clomiphene:DHEA** | 0 | No concerns | Low risk | No concerns | Major concerns | No concerns | Major concerns | **Very low** |
| **Clomiphene:Estrogen** | 0 | No concerns | Low risk | No concerns | Major concerns | No concerns | Major concerns | **Very low** |
| **Clomiphene:GH** | 0 | No concerns | Low risk | No concerns | Major concerns | No concerns | Major concerns | **Very low** |
| **Clomiphene:LH addition** | 0 | No concerns | Low risk | No concerns | Major concerns | No concerns | Major concerns | **Very low** |
| **Clomiphene:Letrozole** | 0 | No concerns | Low risk | No concerns | Major concerns | No concerns | Major concerns | **Very low** |
| **Clomiphene:Testosterone** | 0 | Some concerns | Low risk | No concerns | Major concerns | No concerns | Major concerns | **Very low** |
| **DHEA:Estrogen** | 0 | No concerns | Low risk | No concerns | Major concerns | No concerns | Major concerns | **Very low** |
| **DHEA:GH** | 0 | No concerns | Low risk | No concerns | Major concerns | No concerns | Major concerns | **Very low** |
| **DHEA:LH addition** | 0 | No concerns | Low risk | No concerns | Major concerns | No concerns | Major concerns | **Very low** |
| **DHEA:Letrozole** | 0 | No concerns | Low risk | No concerns | Major concerns | No concerns | Major concerns | **Very low** |
| **DHEA:Testosterone** | 0 | No concerns | Low risk | No concerns | Major concerns | No concerns | Major concerns | **Very low** |
| **Estrogen:GH** | 0 | No concerns | Low risk | No concerns | Major concerns | No concerns | Major concerns | **Very low** |
| **Estrogen:LH addition** | 0 | No concerns | Low risk | No concerns | Major concerns | No concerns | Major concerns | **Very low** |
| **Estrogen:Letrozole** | 0 | No concerns | Low risk | No concerns | Major concerns | No concerns | Major concerns | **Very low** |
| **Estrogen:Testosterone** | 0 | No concerns | Low risk | No concerns | Major concerns | No concerns | Major concerns | **Very low** |
| **GH:LH addition** | 0 | No concerns | Low risk | No concerns | Major concerns | No concerns | Major concerns | **Very low** |
| **GH:Letrozole** | 0 | No concerns | Low risk | No concerns | Major concerns | No concerns | Major concerns | **Very low** |
| **GH:Testosterone** | 0 | No concerns | Low risk | No concerns | Major concerns | No concerns | Major concerns | **Very low** |
| **Letrozole:LH addition** | 0 | No concerns | Low risk | No concerns | Major concerns | No concerns | Major concerns | **Very low** |
| **LH addition:Testosterone** | 0 | No concerns | Low risk | No concerns | Major concerns | No concerns | Major concerns | **Very low** |
| **Letrozole:Testosterone** | 0 | No concerns | Low risk | No concerns | Major concerns | No concerns | Major concerns | **Very low** |

Number of retrieved oocytes

| **Comparison** | **Number of studies** | **Within-study bias** | **Reporting bias** | **Indirectness** | **Imprecision** | **Heterogeneity** | **Incoherence** | **Confidence rating** |
| --- | --- | --- | --- | --- | --- | --- | --- | --- |
| **Clomiphene:Control** | 3 | Some concerns | Low risk | No concerns | Major concerns | No concerns | Major concerns | **Very low** |
| **Control:DHEA** | 4 | No concerns | Low risk | No concerns | Major concerns | No concerns | Major concerns | **Very low** |
| **Control:Estrogen** | 1 | No concerns | Low risk | No concerns | Major concerns | No concerns | Major concerns | **Very low** |
| **Control:GH** | 3 | No concerns | Low risk | No concerns | Major concerns | No concerns | Major concerns | **Very low** |
| **Control:LH addition** | 3 | No concerns | Low risk | No concerns | Major concerns | No concerns | Major concerns | **Very low** |
| **Control:Letrozole** | 3 | No concerns | Low risk | No concerns | Major concerns | No concerns | Major concerns | **Very low** |
| **Control:Testosterone** | 5 | Some concerns | Low risk | No concerns | Major concerns | No concerns | Major concerns | **Very low** |
| **Clomiphene:DHEA** | 0 | No concerns | Low risk | No concerns | Major concerns | No concerns | Major concerns | **Very low** |
| **Clomiphene:Estrogen** | 0 | No concerns | Low risk | No concerns | Major concerns | No concerns | Major concerns | **Very low** |
| **Clomiphene:GH** | 0 | No concerns | Low risk | No concerns | Major concerns | No concerns | Major concerns | **Very low** |
| **Clomiphene:LH addition** | 0 | No concerns | Low risk | No concerns | Major concerns | No concerns | Major concerns | **Very low** |
| **Clomiphene:Letrozole** | 0 | No concerns | Low risk | No concerns | Major concerns | No concerns | Major concerns | **Very low** |
| **Clomiphene:Testosterone** | 0 | Some concerns | Low risk | No concerns | Major concerns | No concerns | Major concerns | **Very low** |
| **DHEA:Estrogen** | 0 | No concerns | Low risk | No concerns | Major concerns | No concerns | Major concerns | **Very low** |
| **DHEA:GH** | 0 | No concerns | Low risk | No concerns | Major concerns | No concerns | Major concerns | **Very low** |
| **DHEA:LH addition** | 0 | No concerns | Low risk | No concerns | Major concerns | No concerns | Major concerns | **Very low** |
| **DHEA:Letrozole** | 0 | No concerns | Low risk | No concerns | Major concerns | No concerns | Major concerns | **Very low** |
| **DHEA:Testosterone** | 0 | No concerns | Low risk | No concerns | Major concerns | No concerns | Major concerns | **Very low** |
| **Estrogen:GH** | 0 | No concerns | Low risk | No concerns | Major concerns | No concerns | Major concerns | **Very low** |
| **Estrogen:LH addition** | 0 | No concerns | Low risk | No concerns | Major concerns | No concerns | Major concerns | **Very low** |
| **Estrogen:Letrozole** | 0 | No concerns | Low risk | No concerns | Major concerns | No concerns | Major concerns | **Very low** |
| **Estrogen:Testosterone** | 0 | No concerns | Low risk | No concerns | Major concerns | No concerns | Major concerns | **Very low** |
| **GH:LH addition** | 0 | No concerns | Low risk | No concerns | Major concerns | No concerns | Major concerns | **Very low** |
| **GH:Letrozole** | 0 | No concerns | Low risk | No concerns | Major concerns | No concerns | Major concerns | **Very low** |
| **GH:Testosterone** | 0 | No concerns | Low risk | No concerns | Major concerns | No concerns | Major concerns | **Very low** |
| **Letrozole:LH addition** | 0 | No concerns | Low risk | No concerns | Major concerns | No concerns | Major concerns | **Very low** |
| **LH addition:Testosterone** | 0 | No concerns | Low risk | No concerns | Major concerns | No concerns | Major concerns | **Very low** |
| **Letrozole:Testosterone** | 0 | No concerns | Low risk | No concerns | Major concerns | No concerns | Major concerns | **Very low** |

Number of retrieved MII oocytes

| **Comparison** | **Number of studies** | **Within-study bias** | **Reporting bias** | **Indirectness** | **Imprecision** | **Heterogeneity** | **Incoherence** | **Confidence rating** |
| --- | --- | --- | --- | --- | --- | --- | --- | --- |
| **Clomiphene:Control** | 2 | Some concerns | Low risk | No concerns | Major concerns | No concerns | Major concerns | **Very low** |
| **Control:DHEA** | 3 | No concerns | Low risk | No concerns | Major concerns | No concerns | Major concerns | **Very low** |
| **Control:Estrogen** | 1 | No concerns | Low risk | No concerns | Major concerns | No concerns | Major concerns | **Very low** |
| **Control:GH** | 2 | No concerns | Low risk | No concerns | No concerns | Major concerns | Major concerns | **Very low** |
| **Control:LH addition** | 3 | No concerns | Low risk | No concerns | Major concerns | No concerns | Major concerns | **Very low** |
| **Control:Letrozole** | 2 | No concerns | Low risk | No concerns | Major concerns | No concerns | Major concerns | **Very low** |
| **Control:Testosterone** | 4 | Some concerns | Low risk | No concerns | Major concerns | No concerns | Major concerns | **Very low** |
| **Clomiphene:DHEA** | 0 | No concerns | Low risk | No concerns | No concerns | Major concerns | Major concerns | **Very low** |
| **Clomiphene:Estrogen** | 0 | No concerns | Low risk | No concerns | Major concerns | No concerns | Major concerns | **Very low** |
| **Clomiphene:GH** | 0 | Some concerns | Low risk | No concerns | No concerns | Major concerns | Major concerns | **Very low** |
| **Clomiphene:LH addition** | 0 | No concerns | Low risk | No concerns | Major concerns | No concerns | Major concerns | **Very low** |
| **Clomiphene:Letrozole** | 0 | No concerns | Low risk | No concerns | Major concerns | No concerns | Major concerns | **Very low** |
| **Clomiphene:Testosterone** | 0 | Some concerns | Low risk | No concerns | Major concerns | No concerns | Major concerns | **Very low** |
| **DHEA:Estrogen** | 0 | No concerns | Low risk | No concerns | Major concerns | No concerns | Major concerns | **Very low** |
| **DHEA:GH** | 0 | No concerns | Low risk | No concerns | Major concerns | No concerns | Major concerns | **Very low** |
| **DHEA:LH addition** | 0 | No concerns | Low risk | No concerns | Major concerns | No concerns | Major concerns | **Very low** |
| **DHEA:Letrozole** | 0 | No concerns | Low risk | No concerns | Major concerns | No concerns | Major concerns | **Very low** |
| **DHEA:Testosterone** | 0 | No concerns | Low risk | No concerns | Major concerns | No concerns | Major concerns | **Very low** |
| **Estrogen:GH** | 0 | No concerns | Low risk | No concerns | Major concerns | No concerns | Major concerns | **Very low** |
| **Estrogen:LH addition** | 0 | No concerns | Low risk | No concerns | Major concerns | No concerns | Major concerns | **Very low** |
| **Estrogen:Letrozole** | 0 | No concerns | Low risk | No concerns | Major concerns | No concerns | Major concerns | **Very low** |
| **Estrogen:Testosterone** | 0 | No concerns | Low risk | No concerns | Major concerns | No concerns | Major concerns | **Very low** |
| **GH:LH addition** | 0 | No concerns | Low risk | No concerns | Major concerns | No concerns | Major concerns | **Very low** |
| **GH:Letrozole** | 0 | No concerns | Low risk | No concerns | Major concerns | No concerns | Major concerns | **Very low** |
| **GH:Testosterone** | 0 | No concerns | Low risk | No concerns | Major concerns | No concerns | Major concerns | **Very low** |
| **Letrozole:LH addition** | 0 | No concerns | Low risk | No concerns | Major concerns | No concerns | Major concerns | **Very low** |
| **LH addition:Testosterone** | 0 | No concerns | Low risk | No concerns | Major concerns | No concerns | Major concerns | **Very low** |
| **Letrozole:Testosterone** | 0 | No concerns | Low risk | No concerns | Major concerns | No concerns | Major concerns | **Very low** |

Estrogen levels at trigger day

| **Comparison** | **Number of studies** | **Within-study bias** | **Reporting bias** | **Indirectness** | **Imprecision** | **Heterogeneity** | **Incoherence** | **Confidence rating** |
| --- | --- | --- | --- | --- | --- | --- | --- | --- |
| **Clomiphene:Control** | 1 | No concerns | Low risk | No concerns | Major concerns | No concerns | Major concerns | **Very low** |
| **Control:DHEA** | 3 | No concerns | Low risk | No concerns | Major concerns | No concerns | Major concerns | **Very low** |
| **Control:Estrogen** | 1 | No concerns | Low risk | No concerns | Major concerns | No concerns | Major concerns | **Very low** |
| **Control:GH** | 2 | No concerns | Low risk | No concerns | Major concerns | No concerns | Major concerns | **Very low** |
| **Control:LH addition** | 2 | No concerns | Low risk | No concerns | Major concerns | No concerns | Major concerns | **Very low** |
| **Control:Letrozole** | 2 | No concerns | Low risk | No concerns | No concerns | Major concerns | Major concerns | **Very low** |
| **Control:Testosterone** | 3 | Some concerns | Low risk | No concerns | Major concerns | No concerns | Major concerns | **Very low** |
| **Clomiphene:DHEA** | 0 | No concerns | Low risk | No concerns | Major concerns | No concerns | Major concerns | **Very low** |
| **Clomiphene:Estrogen** | 0 | No concerns | Low risk | No concerns | Major concerns | No concerns | Major concerns | **Very low** |
| **Clomiphene:GH** | 0 | No concerns | Low risk | No concerns | Major concerns | No concerns | Major concerns | **Very low** |
| **Clomiphene:LH addition** | 0 | No concerns | Low risk | No concerns | Major concerns | No concerns | Major concerns | **Very low** |
| **Clomiphene:Letrozole** | 0 | No concerns | Low risk | No concerns | No concerns | Major concerns | Major concerns | **Very low** |
| **Clomiphene:Testosterone** | 0 | No concerns | Low risk | No concerns | Major concerns | No concerns | Major concerns | **Very low** |
| **DHEA:Estrogen** | 0 | No concerns | Low risk | No concerns | Major concerns | No concerns | Major concerns | **Very low** |
| **DHEA:GH** | 0 | No concerns | Low risk | No concerns | Major concerns | No concerns | Major concerns | **Very low** |
| **DHEA:LH addition** | 0 | No concerns | Low risk | No concerns | Major concerns | No concerns | Major concerns | **Very low** |
| **DHEA:Letrozole** | 0 | No concerns | Low risk | No concerns | Major concerns | No concerns | Major concerns | **Very low** |
| **DHEA:Testosterone** | 0 | No concerns | Low risk | No concerns | Major concerns | No concerns | Major concerns | **Very low** |
| **Estrogen:GH** | 0 | No concerns | Low risk | No concerns | Major concerns | No concerns | Major concerns | **Very low** |
| **Estrogen:LH addition** | 0 | No concerns | Low risk | No concerns | Major concerns | No concerns | Major concerns | **Very low** |
| **Estrogen:Letrozole** | 0 | No concerns | Low risk | No concerns | Major concerns | No concerns | Major concerns | **Very low** |
| **Estrogen:Testosterone** | 0 | No concerns | Low risk | No concerns | Major concerns | No concerns | Major concerns | **Very low** |
| **GH:LH addition** | 0 | No concerns | Low risk | No concerns | Major concerns | No concerns | Major concerns | **Very low** |
| **GH:Letrozole** | 0 | No concerns | Low risk | No concerns | No concerns | Major concerns | Major concerns | **Very low** |
| **GH:Testosterone** | 0 | No concerns | Low risk | No concerns | Major concerns | No concerns | Major concerns | **Very low** |
| **Letrozole:LH addition** | 0 | No concerns | Low risk | No concerns | No concerns | Major concerns | Major concerns | **Very low** |
| **LH addition:Testosterone** | 0 | No concerns | Low risk | No concerns | Major concerns | No concerns | Major concerns | **Very low** |
| **Letrozole:Testosterone** | 0 | No concerns | Low risk | No concerns | No concerns | Major concerns | Major concerns | **Very low** |

Total dose of gonadotropins

| **Comparison** | **Number of studies** | **Within-study bias** | **Reporting bias** | **Indirectness** | **Imprecision** | **Heterogeneity** | **Incoherence** | **Confidence rating** |
| --- | --- | --- | --- | --- | --- | --- | --- | --- |
| **Clomiphene:Control** | 2 | No concerns | Low risk | No concerns | Major concerns | No concerns | Major concerns | **Very low** |
| **Control:DHEA** | 2 | No concerns | Low risk | No concerns | Major concerns | No concerns | Major concerns | **Very low** |
| **Control:Estrogen** | 1 | No concerns | Low risk | No concerns | Major concerns | No concerns | Major concerns | **Very low** |
| **Control:GH** | 2 | Major concerns | Low risk | No concerns | Major concerns | No concerns | Major concerns | **Very low** |
| **Control:LH addition** | 3 | No concerns | Low risk | No concerns | Major concerns | No concerns | Major concerns | **Very low** |
| **Control:Letrozole** | 3 | No concerns | Low risk | No concerns | No concerns | No concerns | Major concerns | **Low** |
| **Control:Testosterone** | 2 | Some concerns | Low risk | No concerns | Major concerns | No concerns | Major concerns | **Very low** |
| **Clomiphene:DHEA** | 0 | No concerns | Low risk | No concerns | Major concerns | No concerns | Major concerns | **Very low** |
| **Clomiphene:Estrogen** | 0 | No concerns | Low risk | No concerns | Major concerns | No concerns | Major concerns | **Very low** |
| **Clomiphene:GH** | 0 | No concerns | Low risk | No concerns | Major concerns | No concerns | Major concerns | **Very low** |
| **Clomiphene:LH addition** | 0 | No concerns | Low risk | No concerns | Major concerns | No concerns | Major concerns | **Very low** |
| **Clomiphene:Letrozole** | 0 | No concerns | Low risk | No concerns | No concerns | Major concerns | Major concerns | **Very low** |
| **Clomiphene:Testosterone** | 0 | No concerns | Low risk | No concerns | Major concerns | No concerns | Major concerns | **Very low** |
| **DHEA:Estrogen** | 0 | No concerns | Low risk | No concerns | Major concerns | No concerns | Major concerns | **Very low** |
| **DHEA:GH** | 0 | No concerns | Low risk | No concerns | Major concerns | No concerns | Major concerns | **Very low** |
| **DHEA:LH addition** | 0 | No concerns | Low risk | No concerns | Major concerns | No concerns | Major concerns | **Very low** |
| **DHEA:Letrozole** | 0 | No concerns | Low risk | No concerns | No concerns | No concerns | Major concerns | **Low** |
| **DHEA:Testosterone** | 0 | No concerns | Low risk | No concerns | Major concerns | No concerns | Major concerns | **Very low** |
| **Estrogen:GH** | 0 | No concerns | Low risk | No concerns | Major concerns | No concerns | Major concerns | **Very low** |
| **Estrogen:LH addition** | 0 | No concerns | Low risk | No concerns | Major concerns | No concerns | Major concerns | **Very low** |
| **Estrogen:Letrozole** | 0 | No concerns | Low risk | No concerns | No concerns | No concerns | Major concerns | **Low** |
| **Estrogen:Testosterone** | 0 | No concerns | Low risk | No concerns | Major concerns | No concerns | Major concerns | **Very low** |
| **GH:LH addition** | 0 | No concerns | Low risk | No concerns | Major concerns | No concerns | Major concerns | **Very low** |
| **GH:Letrozole** | 0 | No concerns | Low risk | No concerns | No concerns | No concerns | Major concerns | **Low** |
| **GH:Testosterone** | 0 | No concerns | Low risk | No concerns | Major concerns | No concerns | Major concerns | **Very low** |
| **Letrozole:LH addition** | 0 | No concerns | Low risk | No concerns | No concerns | No concerns | Major concerns | **Low** |
| **LH addition:Testosterone** | 0 | No concerns | Low risk | No concerns | Major concerns | No concerns | Major concerns | **Very low** |
| **Letrozole:Testosterone** | 0 | No concerns | Low risk | No concerns | No concerns | No concerns | Major concerns | **Low** |

Duration of COS

| **Comparison** | **Number of studies** | **Within-study bias** | **Reporting bias** | **Indirectness** | **Imprecision** | **Heterogeneity** | **Incoherence** | **Confidence rating** |
| --- | --- | --- | --- | --- | --- | --- | --- | --- |
| **Clomiphene:Control** | 2 | No concerns | Low risk | No concerns | Major concerns | No concerns | Major concerns | **Very low** |
| **Control:DHEA** | 2 | No concerns | Low risk | No concerns | Major concerns | No concerns | Major concerns | **Very low** |
| **Control:Estrogen** | 1 | No concerns | Low risk | No concerns | Major concerns | No concerns | Major concerns | **Very low** |
| **Control:GH** | 3 | No concerns | Low risk | No concerns | Major concerns | No concerns | Major concerns | **Very low** |
| **Control:LH addition** | 1 | No concerns | Low risk | No concerns | Major concerns | No concerns | Major concerns | **Very low** |
| **Control:Letrozole** | 3 | No concerns | Low risk | No concerns | No concerns | Major concerns | Major concerns | **Very low** |
| **Control:Testosterone** | 3 | Some concerns | Low risk | No concerns | Major concerns | No concerns | Major concerns | **Very low** |
| **Clomiphene:DHEA** | 0 | No concerns | Low risk | No concerns | Major concerns | No concerns | Major concerns | **Very low** |
| **Clomiphene:Estrogen** | 0 | No concerns | Low risk | No concerns | Major concerns | No concerns | Major concerns | **Very low** |
| **Clomiphene:GH** | 0 | No concerns | Low risk | No concerns | Major concerns | No concerns | Major concerns | **Very low** |
| **Clomiphene:LH addition** | 0 | No concerns | Low risk | No concerns | Major concerns | No concerns | Major concerns | **Very low** |
| **Clomiphene:Letrozole** | 0 | No concerns | Low risk | No concerns | Major concerns | No concerns | Major concerns | **Very low** |
| **Clomiphene:Testosterone** | 0 | Some concerns | Low risk | No concerns | Major concerns | No concerns | Major concerns | **Very low** |
| **DHEA:Estrogen** | 0 | No concerns | Low risk | No concerns | Major concerns | No concerns | Major concerns | **Very low** |
| **DHEA:GH** | 0 | No concerns | Low risk | No concerns | Major concerns | No concerns | Major concerns | **Very low** |
| **DHEA:LH addition** | 0 | No concerns | Low risk | No concerns | Major concerns | No concerns | Major concerns | **Very low** |
| **DHEA:Letrozole** | 0 | No concerns | Low risk | No concerns | Major concerns | No concerns | Major concerns | **Very low** |
| **DHEA:Testosterone** | 0 | No concerns | Low risk | No concerns | Major concerns | No concerns | Major concerns | **Very low** |
| **Estrogen:GH** | 0 | No concerns | Low risk | No concerns | Major concerns | No concerns | Major concerns | **Very low** |
| **Estrogen:LH addition** | 0 | No concerns | Low risk | No concerns | Major concerns | No concerns | Major concerns | **Very low** |
| **Estrogen:Letrozole** | 0 | No concerns | Low risk | No concerns | Major concerns | No concerns | Major concerns | **Very low** |
| **Estrogen:Testosterone** | 0 | No concerns | Low risk | No concerns | Major concerns | No concerns | Major concerns | **Very low** |
| **GH:LH addition** | 0 | No concerns | Low risk | No concerns | Major concerns | No concerns | Major concerns | **Very low** |
| **GH:Letrozole** | 0 | No concerns | Low risk | No concerns | Major concerns | No concerns | Major concerns | **Very low** |
| **GH:Testosterone** | 0 | No concerns | Low risk | No concerns | Major concerns | No concerns | Major concerns | **Very low** |
| **Letrozole:LH addition** | 0 | No concerns | Low risk | No concerns | Major concerns | No concerns | Major concerns | **Very low** |
| **LH addition:Testosterone** | 0 | No concerns | Low risk | No concerns | Major concerns | No concerns | Major concerns | **Very low** |
| **Letrozole:Testosterone** | 0 | No concerns | Low risk | No concerns | Major concerns | No concerns | Major concerns | **Very low** |
