# Supplementary figures and images for: Effectiveness of hormone add-on strategies in ovarian stimulation for women with poor ovarian response: a systematic review and network meta-analysis of randomized controlled trials
Source: J Assist Reprod Genet. 2025 Oct 25;42(10):3231–52. doi: 10.1007/s10815-025-03633-z (PMC12602825; doi:10.1007/s10815-025-03633-z)

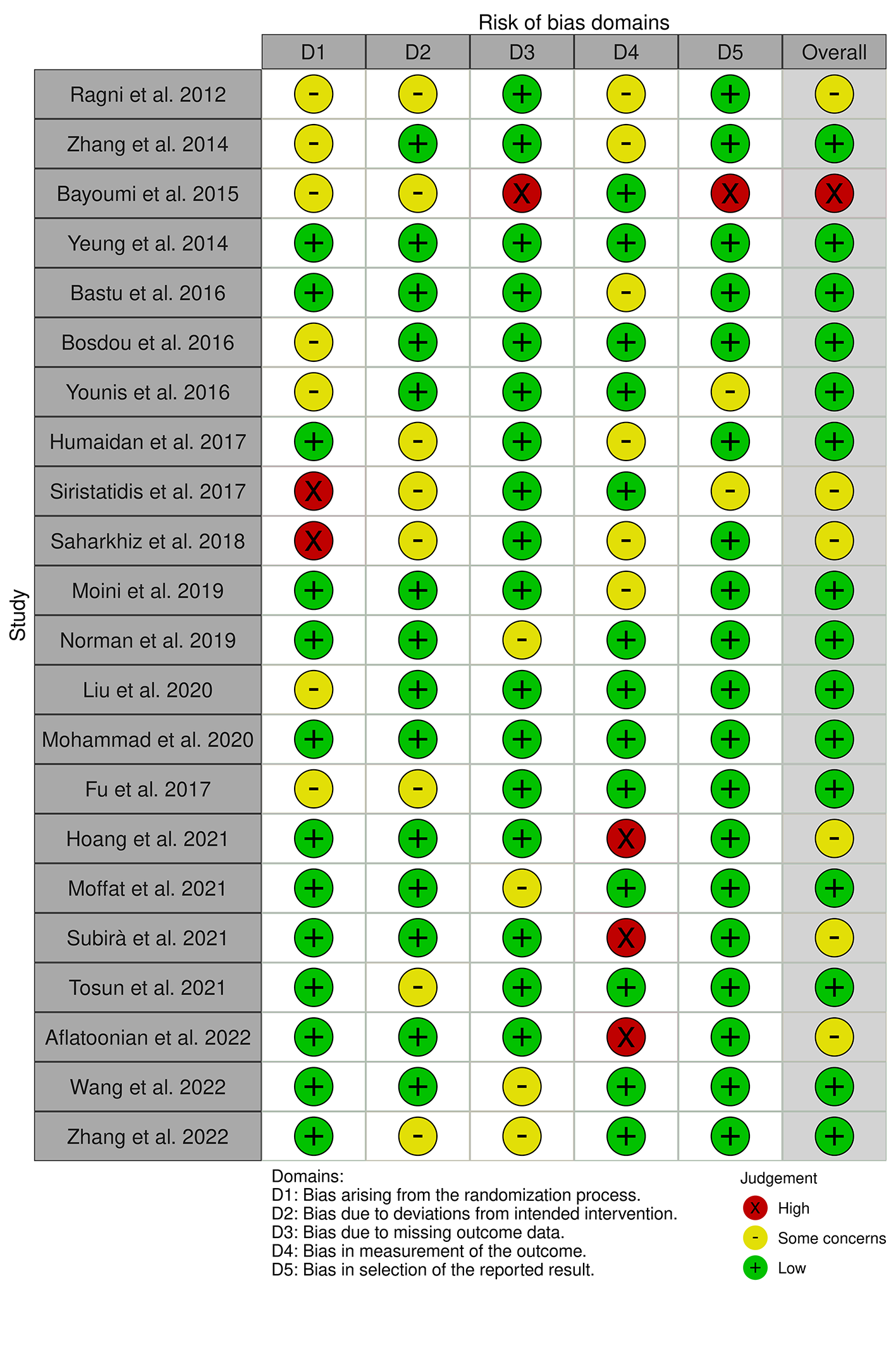

Supplement: Supplementary file 3 — (PNG 621 KB) [file 10815_2025_3633_Fig6_ESM.png]

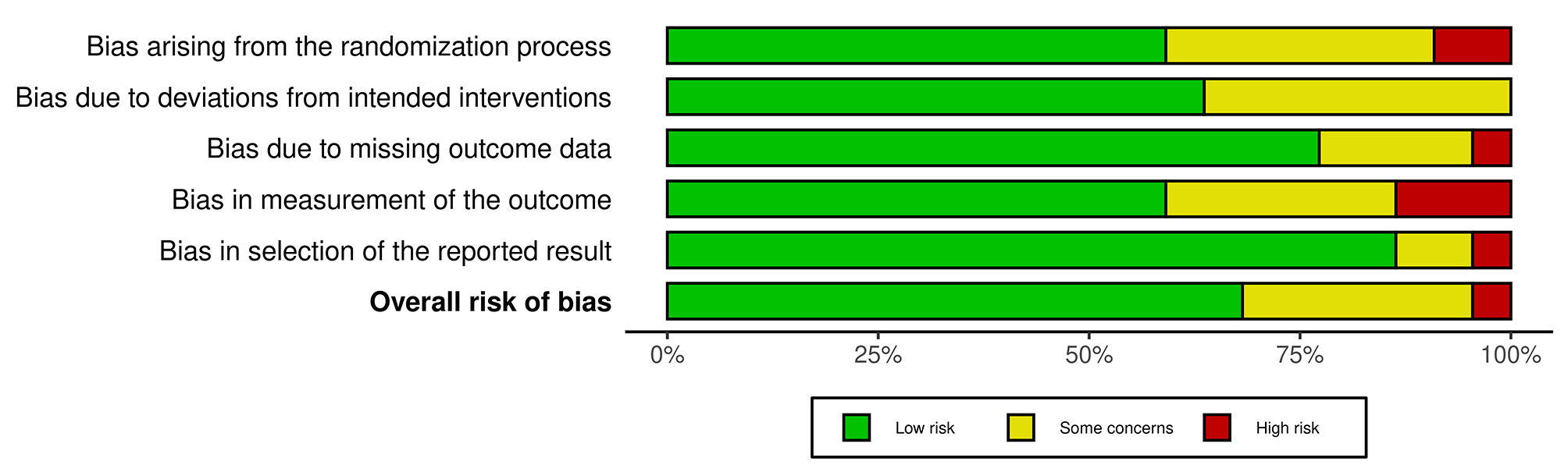

Supplement: Supplementary file 4 — (PNG 83.9 KB) [file 10815_2025_3633_Fig7_ESM.png]

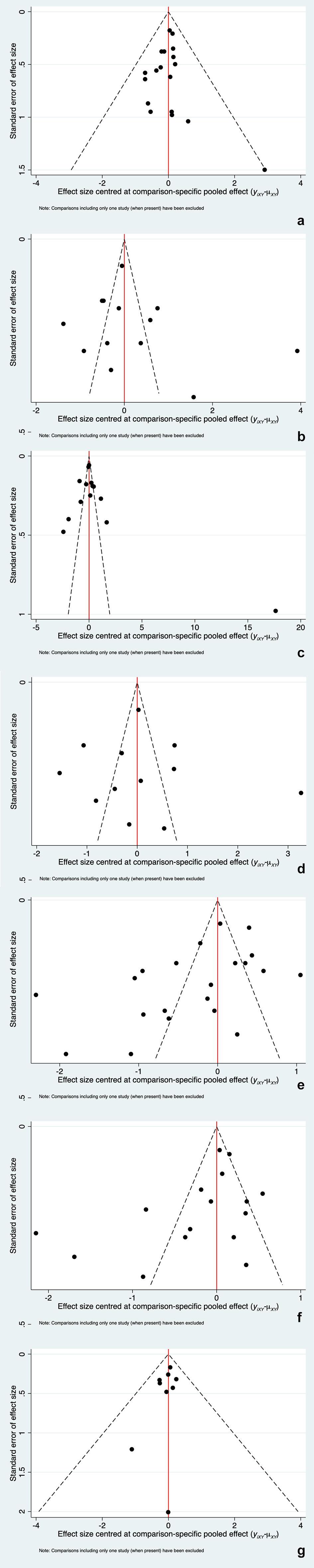

Supplement: Supplementary file 5 — (PNG 481 KB) [file 10815_2025_3633_Fig8_ESM.png]

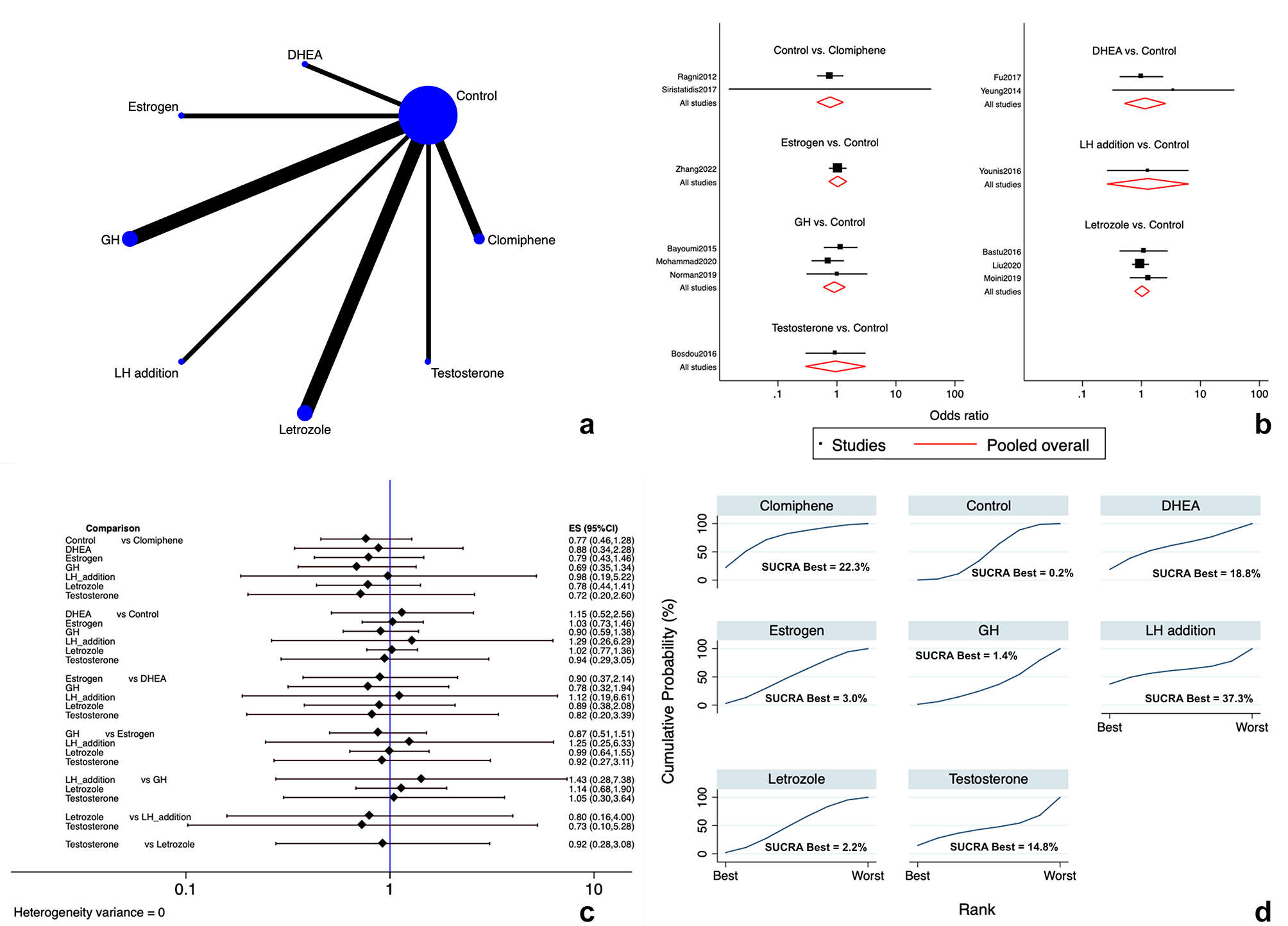

Supplement: Supplementary file 6 — (PNG 376 KB) [file 10815_2025_3633_Fig9_ESM.png]

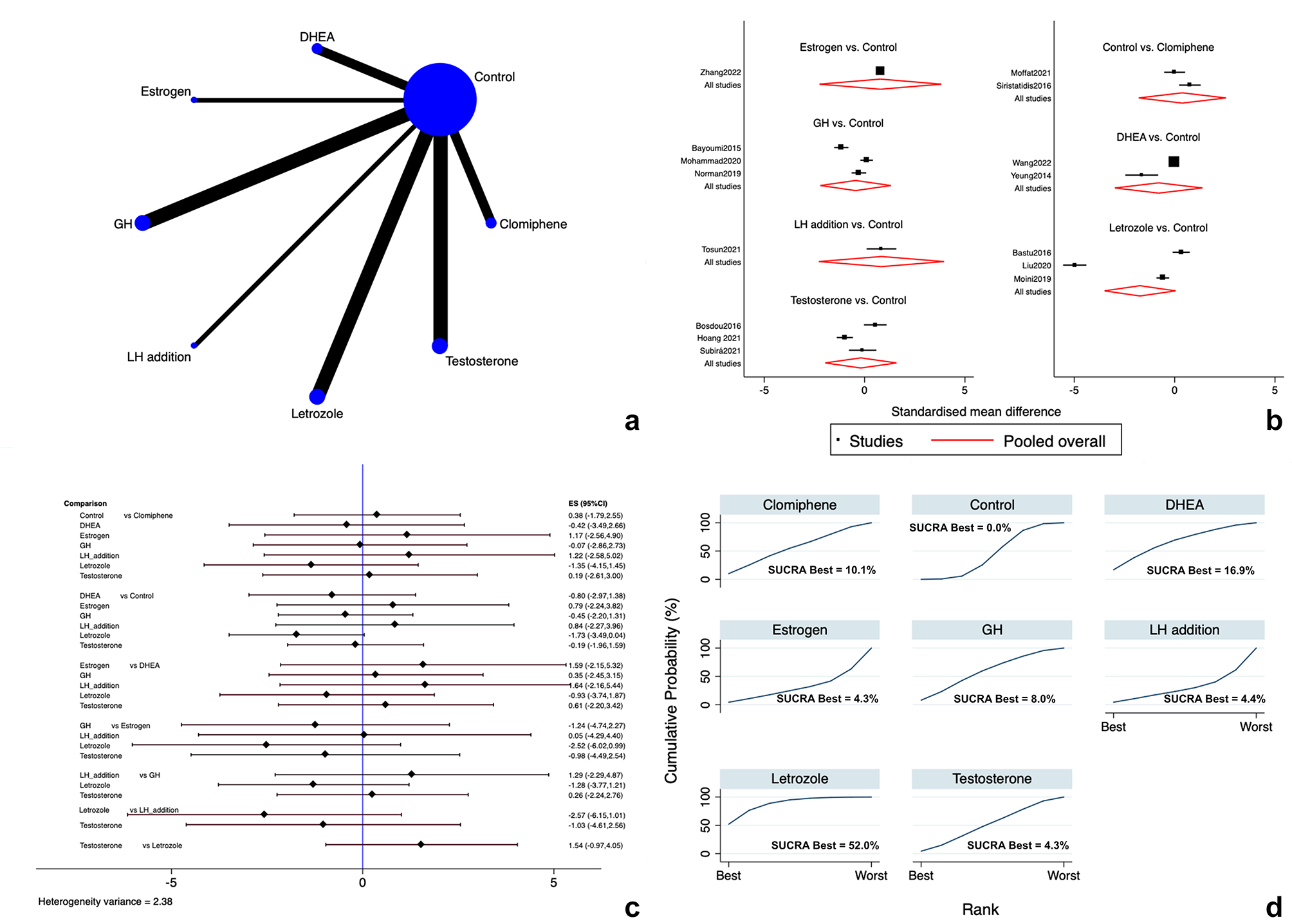

Supplement: Supplementary file 7 — (PNG 362 KB) [file 10815_2025_3633_Fig10_ESM.png]

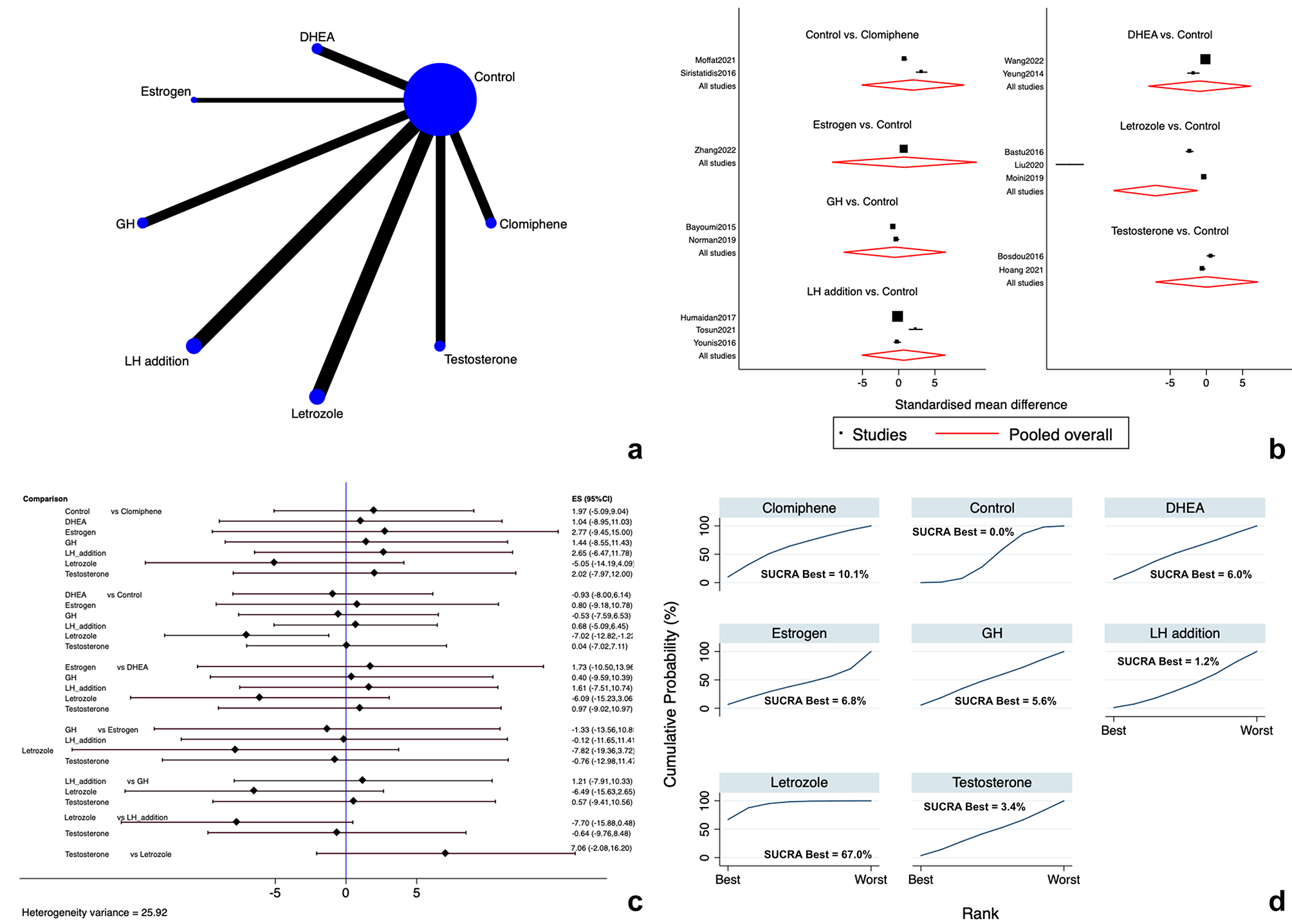

Supplement: Supplementary file 8 — (PNG 376 KB) [file 10815_2025_3633_Fig11_ESM.png]

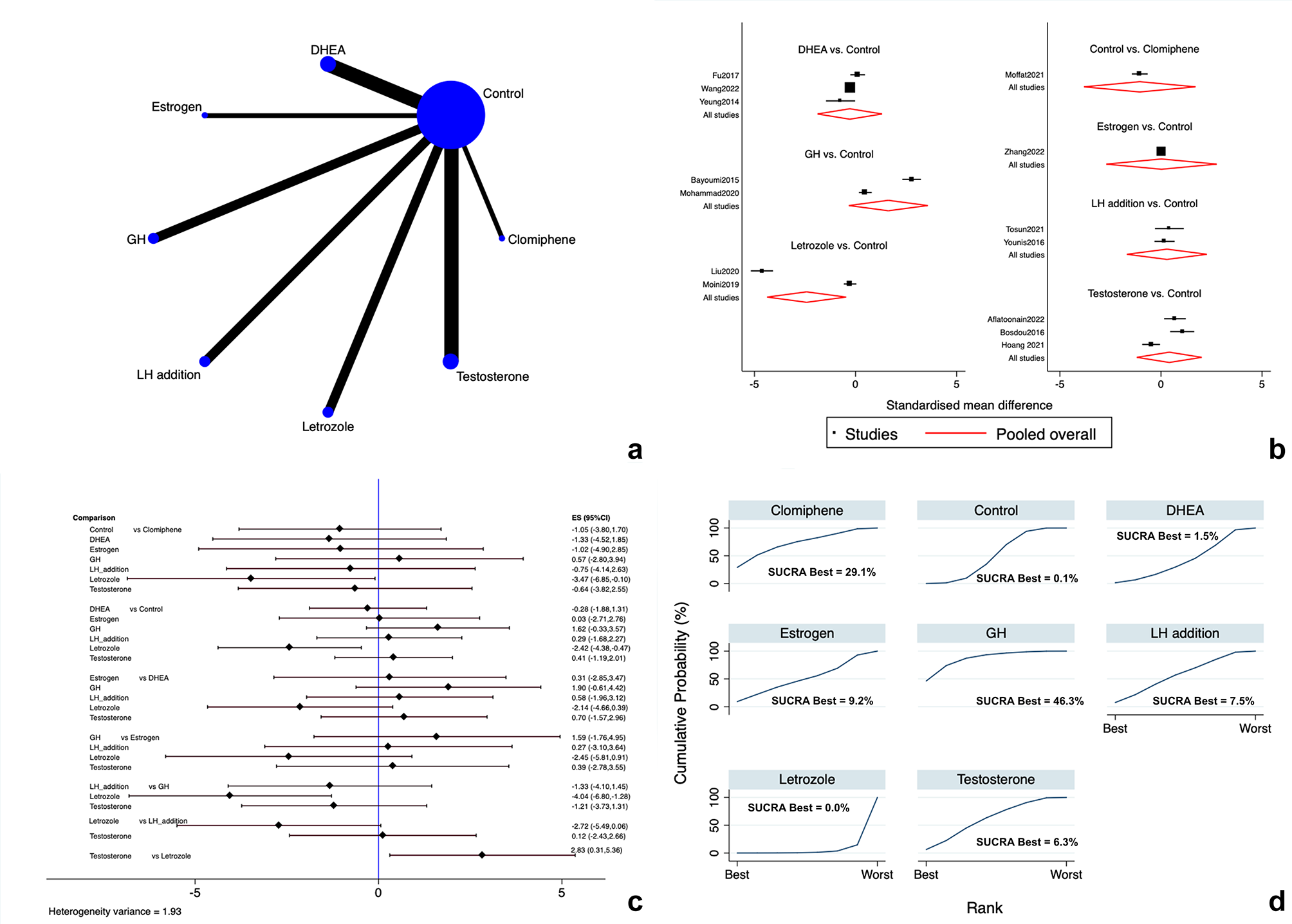

Supplement: Supplementary file 9 — (PNG 356 KB) [file 10815_2025_3633_Fig12_ESM.png]

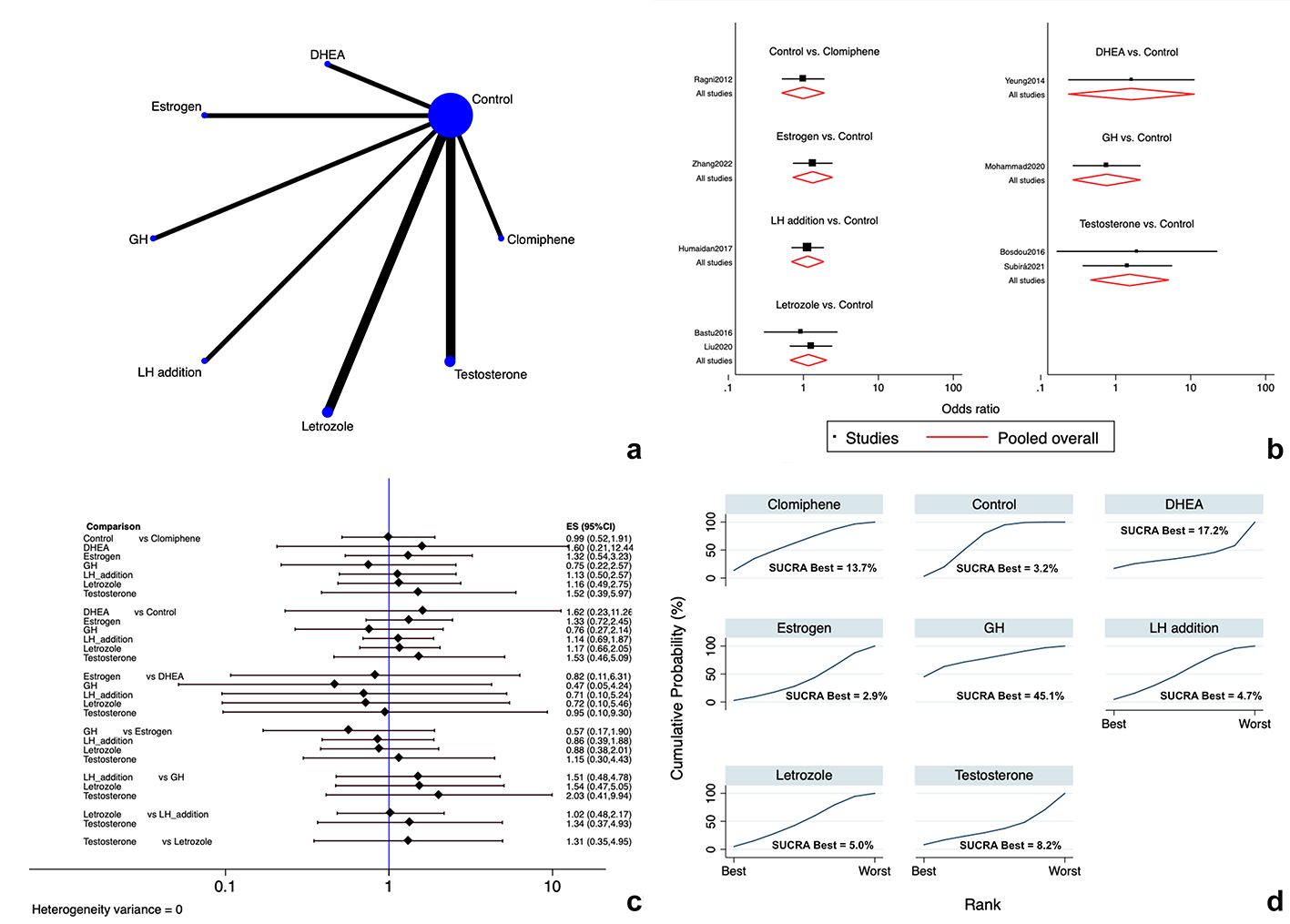

Supplement: Supplementary file 10 — (PNG 213 KB) [file 10815_2025_3633_Fig13_ESM.png]
